# CRISPRa-mediated disentanglement of the Dux-MERVL axis in the 2C-like state, totipotency, and cell death

**DOI:** 10.1126/sciadv.adu9092

**Published:** 2025-12-19

**Authors:** Paul Chammas, Sheila Q. Xie, Lessly P. Sepulveda-Rincon, Bryony J. Leeke, Marian H. Dore, Dirk Dormann, Ryan T. Wagner, Ning Chang, Peter L. Jones, Michael T. McManus, Mohammad M. Karimi, George Young, Michelle Percharde

**Affiliations:** ^1^ MRC Laboratory of Medical Sciences, London W12 0HS, UK.; ^2^ Institute of Clinical Sciences, Imperial College, London W12 0HS, UK.; ^3^ University of California at San Francisco, San Francisco, CA 91413, USA.; ^4^ Department of Pharmacology, University of Nevada, Reno School of Medicine, Reno, NV 89557, USA.

## Abstract

Transposable elements (TEs) are powerful cis-regulatory drivers of gene expression, particularly during early development when many TEs become de-repressed. MERVL elements are transiently up-regulated in mouse totipotent two-cell (2C) embryos during major zygotic genome activation (ZGA) and 2C-like cells in vitro. One of the most powerful activators of MERVL is the pioneer transcription factor, Dux. However, apparent differences lie in the requirement for Dux versus MERVL activation in embryos. Moreover, sustained Dux activation causes cell toxicity, which may or may not be linked to MERVL. Using a CRISPR activation system, we unpick the relative role of Dux and MERVL in ZGA, totipotent-like characteristics, and cell toxicity. We find that MERVL activation drives a portion of the Dux-dependent transcriptome, sufficient for expanded fate potential, but not other totipotency features. Conversely, Dux-induced pathology is independent of MERVL activation and involves the proapoptotic factor, Noxa. Our study highlights the complexity of the Dux-MERVL transcriptional network and uncovers a previously unknown player in Dux-driven pathology.

## Teaser

CRISPRa reveals separable regulatory axes and molecular drivers of the two-cell like state.

## INTRODUCTION

Transposable elements (TEs) are repetitive, genomic sequences that are either historically or now mobile within our DNA. Of these, endogenous retrovirus (ERV) sequences make up ~8 to 10% of mammalian genomes and have arisen from ancient viral infections. While TE activation can be harmful—driving DNA damage, mutagenesis, or inflammation (*1*–*4*)—TE-mediated spread of cis-regulatory elements has played a powerful role in gene expression networks (*5*, *6*). In particular, the long terminal repeat (LTR) promoters of ERVs may harbor transcription factor binding sites and act as alternative promoters or enhancers for nearby genes (*7*–*10*).

Although repressed in most somatic contexts, specific families of TEs are up-regulated in early embryos (*11*–*14*). In mice, murine endogenous retrovirus-L (MERVL) elements are highly and specifically activated in two-cell (2C) stage embryos and then rapidly repressed upon 2C exit (*15*–*19*). The MERVL LTR (MT2_Mm) acts as a powerful promoter for nearby genes and drives activation of 2C-specific genes in embryos (*15*, *20*). The activation of MERVL or the 2C program has been suggested to be essential for zygotic genome activation (ZGA) and embryo progression (*21*–*23*). Transient MERVL activation is also seen in rare cells arising from cultures of embryonic stem cells (ESCs), which express 2C-specific genes and TEs (*16*, *24*). Moreover, these cells, termed 2C-like cells, display other markers of 2C embryos such as increased chromatin accessibility and down-regulation of pluripotency factor expression (*20*, *24*–*26*). They also show totipotent-like characteristics: an ability to contribute to extraembryonic lineages in chimeras (*16*, *27*, *28*). These data point to an integral role for MERVL in the 2C embryo and the 2C-like state.

Despite this, the mechanism of MERVL up-regulation, as well as its exact role in ZGA, is still unclear. In ESCs, the homeobox transcription factor Dux has been found to potently activate MERVL elements and induce the 2C-like state (*29*–*31*). Its human homolog, DUX4, also activates HERVL, MLT2A1, and MLT2A2 TEs during human ZGA at the four- to eight-cell stage (*31*, *32*). However, in vivo, Dux knockout (KO) impedes development but is ultimately viable (*33*–*35*), with other factors presumably sustaining MERVL and 2C/ZGA gene expression (*36*–*38*). Conversely, failure to shut down expression of Dux/DUX4 and TE expression in various cell and embryo models has been associated with DNA damage, cell death, or developmental arrest (*13*, *32*, *35*, *39*, *40*). DUX4 de-repression in muscle cells moreover drives cell death and the human disease, facioscapulohumeral muscular dystrophy (FSHD) (*41*, *42*). Thus, Dux/DUX4 must be tightly controlled in normal cells. This raises the question—which parts of the Dux-MERVL axis are most important for development and 2C characteristics? In addition, are the negative consequences of Dux overexpression due to MERVL activation or independent of TE up-regulation?

Here, we assess both the beneficial and pathological contributions of Dux and MERVL activation in ESCs and embryos. With CRISPR activation (CRISPRa) and our recently described 2C–green fluorescent protein (GFP)/CD4 reporter ESCs (*40*), we show that direct Dux activation much more faithfully resembles endogenous 2C-like cells than 2C-like cells driven by MERVL alone. However, despite MERVL only activating a portion of 2C-like genes and features, it is equally efficient in conferring expanded fate potential. Conversely, Dux, but not MERVL, is associated with reduced proliferation and cell death and involves activation of the proapoptotic factor, Noxa. Our study places MERVL as a partial yet key feature of the 2C-like state and reveals its separation from the pathological impacts of Dux overexpression.

## RESULTS

### Generation of a 2C-GFP/CD4 CRISPRa system

**Fig. 1.**
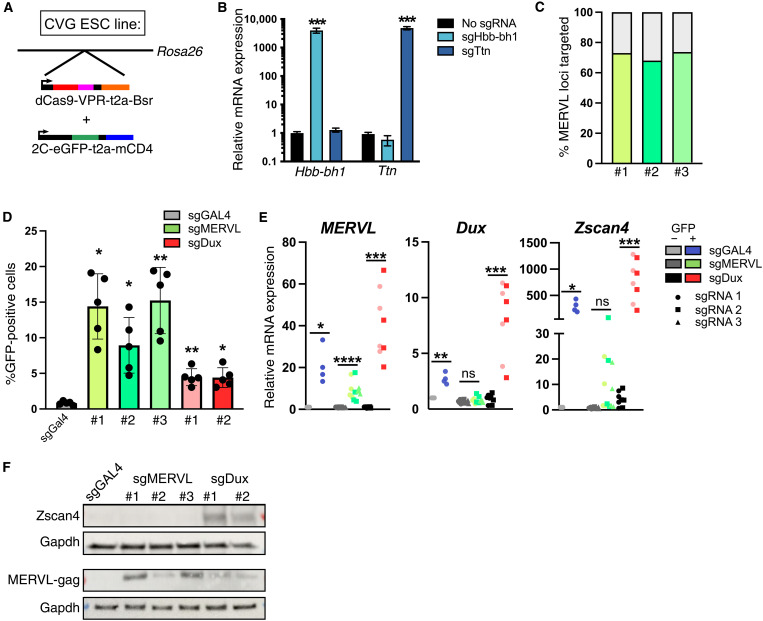
Establishment of a CRISPRa system for 2C-like cell generation. (**A**) Diagram of CVG ESCs: a single clone of E14 ESCs targeted with dCas9-VPR into the *Rosa* locus and then a 2C-GFP/CD4 construct by random integration. (**B**) RT-qPCR validation of CRISPRa by dCas9-VPR in ESCs transduced with the indicated sgRNAs. Data are mean ± SEM of three independent Rosa^dCas9-VPR/+^ clones. ***P < 0.001, two-tailed Student’s *t* test. (**C**) Predicted binding of the indicated sgRNAs to MERVL MT2_Mm loci. (**D**) Flow cytometry analysis of the percentage of GFP-positive cells in CVG ESCs 48 hours after transfection with the indicated sgRNAs. Data are mean ± SEM of *n* = 5 independent experiments. *P < 0.05 and **P < 0.01, one-way Welch’s analysis of variance (ANOVA) comparing each condition to sgGAL4, with Brown-Forsyth correction for multiple comparisons. (**E**) RT-qPCR analysis of the indicated genes in FACS-sorted GFP-negative (−) and positive (+) cells. Data represent *n* = 4 independent experiments. *P < 0.05, **P < 0.01, ***P < 0.001, and ****P < 0.0001, Welch’s unpaired *t* test with Holm-Sidak correction for multiple comparisons. (**F**) Western blot in unsorted CVG ESCs 48 hours after transfection with sgRNAs, representative of three independent experiments. ns, not significant.

To investigate the relationship between Dux and MERVL activation, we used a CRISPRa system combined with our recently published 2C-GFP/CD4 reporter mouse ESCs (*40*). Approximately 1 to 2% of ESCs enter transiently into a 2C-like state, termed 2C-like cells, which can be identified by MERVL-driven GFP expression and purified by fluorescence-activated cell sorting (FACS) or CD4-based magnetic isolation. These ESCs were targeted with a dead Cas9 construct fused to the tripartite VP16-p16-RelA activator [dCas9-VPR (CV)] (*43*) into the *Rosa26* locus (Fig. 1A and fig. S1, A and B). We confirmed the functionality of dCas9-VPR by transducing heterozygous *Rosa26^CV/+^* ESCs with single guide RNAs (sgRNAs) targeting two silenced genes, *Hbb-bh1* and *Ttn*, which led to high and specific activation of their relative targets (Fig. 1B). To enable activation of endogenous MERVL loci, we continued with a single *Rosa26^CV/+^*, 2C-GFP/CD4 clone for all subsequent experiments (fig. S1B), referred to subsequently as CVG ESCs. Three independent sgRNAs were selected that target the MERVL consensus sequence and were each predicted to match 65 to 70% of individual MERVL copies (Fig. 1C, fig. S1C, and table S1). Transient transfection of CVG ESCs with these guides alongside two Dux sgRNAs (fig. S1D) or a negative control guide (sgGAL4) resulted in a robust increase in 2C-GFP–positive cells compared to the control (Fig. 1D). Note that sgGAL4 is not predicted to bind to any mouse genes, so 2C-like cells arising from this transfection are very rare and represent spontaneously occurring, or endogenous, 2C-like cells. We next validated up-regulation of *Dux* or MERVL RNA by quantitative reverse transcription polymerase chain reaction (RT-qPCR). MERVL expression is induced in GFP-positive cells from all three sgRNA transfections (Fig. 1, E and F). In contrast, *Dux* is activated only in sgGAL4 and sgDux cells and not sgMERVL. This is consistent with MERVL elements as downstream of Dux. *Zscan4* is interestingly not activated by sgMERVL, suggesting it to be a direct Dux target (Fig. 1, E and F). These data confirm the establishment of an efficient system to activate endogenous *Dux* or MERVL in ESCs and begin to point to differences between the 2C-like cells they induce.

### MERVL CRISPRa generates an intermediate 2C-like transcriptome

**Fig. 2.**
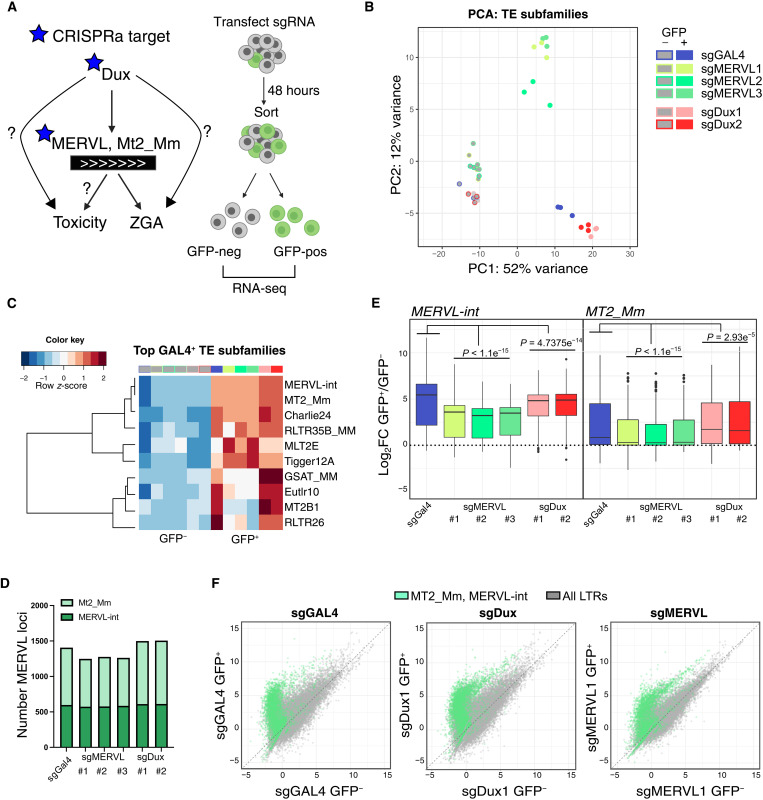
Activation of TEs by Dux- or MERVL-induced 2C-like cells. (**A**) Diagram of CRISPRa experiments and questions. sgRNAs against Dux or MERVL elements were used to probe the relative contribution of distinct portions of this axis to ZGA and to cell toxicity. GFP-positive/negative cells were FACS-purified from sgRNA-transfected CVG ESCs. (**B**) PCA of TE subfamily expression in GFP-negative (−) and GFP-positive (+) cells from the indicated sgRNA transfections. (**C**) Heatmap showing the expression of the top 10 most up-regulated TE subfamilies in control 2C-like cells (sgGAL4-GFP^+^ versus GFP^−^ cells) across all conditions. (**D**) Histogram of the number of significantly up-regulated MT2_Mm and MERVL-int loci in GFP-positive, 2C-like cells compared to their GFP-negative control [false discovery rate (FDR) < 0.05]. (**E**) Boxplot showing log_2_ fold change (log_2_FC) in the expression of individual loci from MERVL-int or MT2_Mm subfamilies between GFP-positive and GFP-negative samples. *P* values, Wilcoxon rank sum test between each condition and sgGAL4, with Bonferroni correction for multiple comparisons. (**F**) Scatterplot showing normalized expression of all individual LTR loci (gray) and MERVL loci (green) in the indicated conditions.

We next set out to probe the global transcriptional impact of Dux and MERVL (Fig. 2A). CVG ESCs were transfected with Dux, MERVL, or GAL4 sgRNAs and pure populations of GFP-positive or GFP-negative cells sorted 48 hours later and processed for RNA sequencing (RNA-seq) (Fig. 2A and table S2). We first looked at the expression of TEs, with clustering of samples based on TE subfamily expression revealing a clear separation between 2C-GFP–positive (2C-like) and 2C-GFP–negative (ESC) samples (Fig. 2B). While GAL4/Dux-induced 2C-like cells cluster together, furthest away from ESCs, MERVL-induced 2C-like cells exhibit an intermediate profile. We examined the top 10 most up-regulated TE subfamilies in endogenous 2C-like cells (sgGAL4, GFP^+^). As expected, MERVL-int and its promoter, MT2_Mm, are activated in all conditions, as well as other ERVL subfamilies such as MT2C_Mm and MLT2E (Fig. 2C and fig. S2, A and B). In contrast, elements such as major satellites (GSAT_MM), and the ERV3 member MT2B1, some of which are also up-regulated in 2C embryos (fig. S2B), are not induced by sgMERVL. These data reveal only a partial TE activation profile upon MERVL CRISPRa.

To further explore TE expression, we investigated individual TE loci (fig. S2C). As expected, MERVL-int and MT2_Mm loci are induced in all GFP-positive samples, with similar numbers of loci significantly activated in each condition (Fig. 2D). While these elements are activated to a similar degree in control versus Dux-induced 2C-like cells, they are consistently induced at slightly lower levels by sgMERVL (Fig. 2, E and F). The MERVL loci induced by individual MERVL sgRNAs are also broadly overlapping (fig. S2D). These data confirm that MERVL CRISPRa effectively activates MERVL expression—but that this forms only a portion of total 2C–up-regulated repeatome.

### Only a portion of 2C-like genes is MERVL dependent

**Fig. 3.**
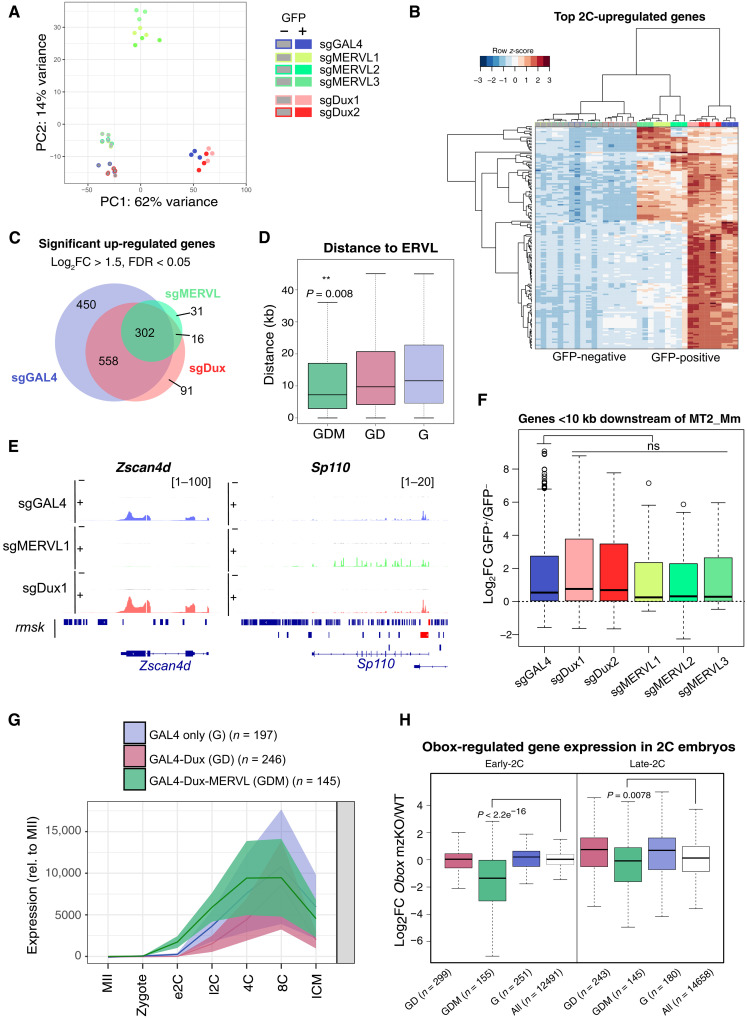
Differential gene induction between MERVL-induced and control 2C-like cells. (**A**) PCA of gene expression in GFP-negative (−) and GFP-positive (+) cells from the indicated sgRNA transfections. (**B**) Heatmap of 2C-like genes [top 200 highest up-regulated genes in control (sgGAL4) 2C-like cells] in all samples. (**C**) Venn diagram indicating the overlap in significantly up-regulated genes in sgGAL4, sgMERVL, and sgDux GFP-positive over GFP-negative cells (log_2_FC > 1.5, FDR < 0.05, in two of two or three sgRNAs in sgDux or sgMERVL experiments, respectively). (**D**) Boxplot showing absolute distances to the nearest ERVL family TE from genes in GDM, GD, or G gene sets. *P* < 0.008, Wilcoxon rank sum test between GDM and G. (**E**) Bigwig plots of the indicated genes in representative GFP-positive (+) and GFP-negative (−) sgRNA samples. MERVL elements are highlighted below the tracks in red. (**F**) Boxplots showing log_2_FC in expression of all genes <10 kb downstream from an MT2_Mm across different conditions. Wilcoxon rank sum test with Bonferroni correction. (**G**) Embryo expression dynamics of genes within the indicated gene sets. Lines and shading depict mean ± SEM gene expression, respectively, relative to MII oocyte. Data from (*46*). (**H**) Boxplots showing log_2_FC in expression of the indicated gene sets in *Obox* mzKO 2C embryos over control embryos. Data from (*36*). *P* values, Wilcoxon rank sum test between GDM genes and all genes.

The LTRs of TEs such as MERVL can act as powerful promoters of nearby genes, and several 2C-specific genes have been confirmed to be driven by a MERVL MT2_Mm promoter (*15*, *23*). We next therefore examined whether partial TE activation is reflected in the differential activation of genes in CRISPRa-driven 2C-like cells. Principal components analysis (PCA) again revealed that MERVL-induced 2C-like cells have an intermediate profile between ESCs and control or Dux-driven 2C-like cells (Fig. 3A). Of the top 150 up-regulated genes in endogenously arising 2C-like cells (GAL4 GFP^+^), i.e., 2C-like genes, ~40% are activated by either MERVL or Dux, while 60% are MERVL independent (Fig. 3B and fig. S3A). In both these cases, Dux-induced 2C-like cells appear, in contrast, very similar to endogenous 2C-like cells. More globally, we found that 558 genes are significantly up-regulated in both GAL4 and Dux-induced 2C-like cells (“GD”) but not MERVL, while 302 genes are also MERVL-activated (“GDM”). Four hundred fifty genes are also induced only in control/endogenous (sgGAL4) 2C-like cells (“G”) (Fig. 3C). Very few genes are activated by sgMERVL alone (*n* = 31), revealing that sgMERVL-activated genes form a subset of Dux targets (Fig. 3C).

A partial induction of the 2C-like transcriptome by MERVL CRISPRa could be due to lower activation of MERVL promoters (Fig. 2E), or because many 2C genes are simply not driven by MERVL. We tested this and investigated the relationship between the MERVL LTR, MT2_Mm, and the gene sets up-regulated. We found that GDM genes are significantly closer to an ERVL element than GD or G gene sets (Fig. 3D). Moreover, 34.8% of GDM genes are within 10 kb of an MT2_Mm, in contrast to only 4.5 or 0.7% of GD or G, respectively (fig. S3B). This suggests that GD genes are not MERVL driven. For example, the 2C gene, *Zscan4d*, is highly up-regulated by Dux alone and has no MT2_Mm within 10 kb. Conversely, *Sp110* is most strongly activated by sgMERVL and is driven by a MT2_Mm element overlapping its promoter (Fig. 3E). We confirmed that lower MERVL activation does not explain the lack of activation of GD genes by sgMERVL, by combining three sgMERVL guides and transfecting increased amounts of sgMERVL (fig. S3C). *Sp110* (GDM) but not *Zscan4/Dux* (GD) is sensitive to MERVL levels (fig. S3C). GDM genes, not GD, are also highly up-regulated in independent MERVL CRISPRa experiments (fig. S3D) (*28*, *44*). Moreover, all genes within 10 kb downstream of an MT2_Mm LTR are efficiently induced by direct MERVL activation at similar or higher levels compared to sgDux (Fig. 3F and fig. S3E). Together, our data reveal that MERVL activation is efficient yet contributes only partially to the 2C transcriptome. In contrast, Dux induces the full spectrum of 2C-like genes, including many non-MERVL LTR-driven targets.

### MERVL-driven genes are Obox dependent in ESCs and embryos

MERVL induction is considered a core feature of the 2C stage in embryos and of the 2C-like state in culture. However, our data show that MERVL activation only partially contributes to the genes up-regulated in 2C-like cells, unlike Dux activation. Nevertheless, Dux has been found to be ultimately dispensable for embryo development, while MERVL depletion causes cleavage stage arrest (*22*, *23*, *33*–*35*). These puzzling discrepancies led us to investigate the functional relevance of MERVL-driven (GDM) or non–MERVL-driven (GD) Dux targets. We first checked expression patterns of these genes in mouse embryos. Postfertilization, all 2C-like gene sets exhibit a general up-regulation, consistent with a possible role in ZGA or as ZGA targets, with GD and GDM genes activated the earliest and being most 2C specific (Fig. 3G and fig. S3F) (*45*, *46*). Using data from dual maternal/zygotic knockout (mzKO) of *Dux*, we also asked whether MERVL-driven or MERVL-independent genes are Dux dependent in vivo. All Dux targets showed negligible gene expression changes upon *Dux* mzKO in zygote and 2C embryos (fig. S4A), consistent with the ultimate viability of *Dux*-deleted embryos. These data suggest that other factors can compensate in vivo for the absence of Dux at both MERVL-dependent and MERVL-independent targets.

We hypothesized that MERVL might act as an integrator of many transcription factors, ensuring MERVL-dependent gene activation at ZGA. We investigated Obox factors, which were recently found to be essential activators of MERVL and ZGA in mice. Obox proteins include both maternally and zygotically expressed isoforms, and *Obox* mzKO leads to down-regulation of MERVL and ZGA genes and causes early embryonic arrest (*36*, *45*). We first confirmed that *Dux* is not a target of Obox in ESCs or embryos (fig. S4B). In contrast, *Obox3/5/6* are up-regulated in control 2C-like cells and by sgDux, but not by sgMERVL (fig. S4C). However, these genes are interestingly not affected in *Dux* mzKO embryos (fig. S4D). Thus, although Dux is sufficient to activate *Obox* isoforms in ESCs, it is not necessary for their expression in embryos.

Next, we investigated whether Obox may collaborate with Dux to activate GDM genes. Using recently published data (*36*), we found that MERVL-driven but not MERVL-independent genes are significantly down-regulated in both early and late *Obox* mzKO 2C embryos (Fig. 3H). Conversely, Obox3/5 overexpression in ESCs directly activates these genes (fig. S4E). These data demonstrate that MERVL-dependent targets are subject to regulation by Obox factors in addition to Dux. Moreover, their down-regulation in *Obox*-deficient embryos before their arrest could suggest an important role of GDM genes in ZGA. In contrast, Dux-driven MERVL-independent genes do not change upon *Obox* mzKO. These genes are therefore potentially less important in early embryos. These data also suggest that Dux alone, unlike Obox, is not sufficient to maintain expression of MERVL-dependent genes in 2C embryos.

We further investigated the developmental relevance of MERVL-driven genes. GDM genes are also more highly down-regulated in somatic cell nuclear transfer (SCNT) experiments compared to intracytoplasmic sperm injection (associated with better embryo development) and significantly up-regulated upon *Obox3* injection, which rescues developmental competence (fig. S5A) (*45*). To compare the relationship between different gene sets and ZGA, we also took advantage of a recent single-cell RNA-seq–based CRISPRa screen (*44*). Integrating data from 10 potential inducers of ZGA-like expression revealed GDM gene activation to be most closely correlated with a ZGA-like response (fig. S5B). Overall, these results point to MERVL and MERVL-driven genes as integrators of multiple transcription factors, which are associated with developmental competence and ZGA.

### Dux and MERVL-driven 2C-like cells show different totipotency features

**Fig. 4.**
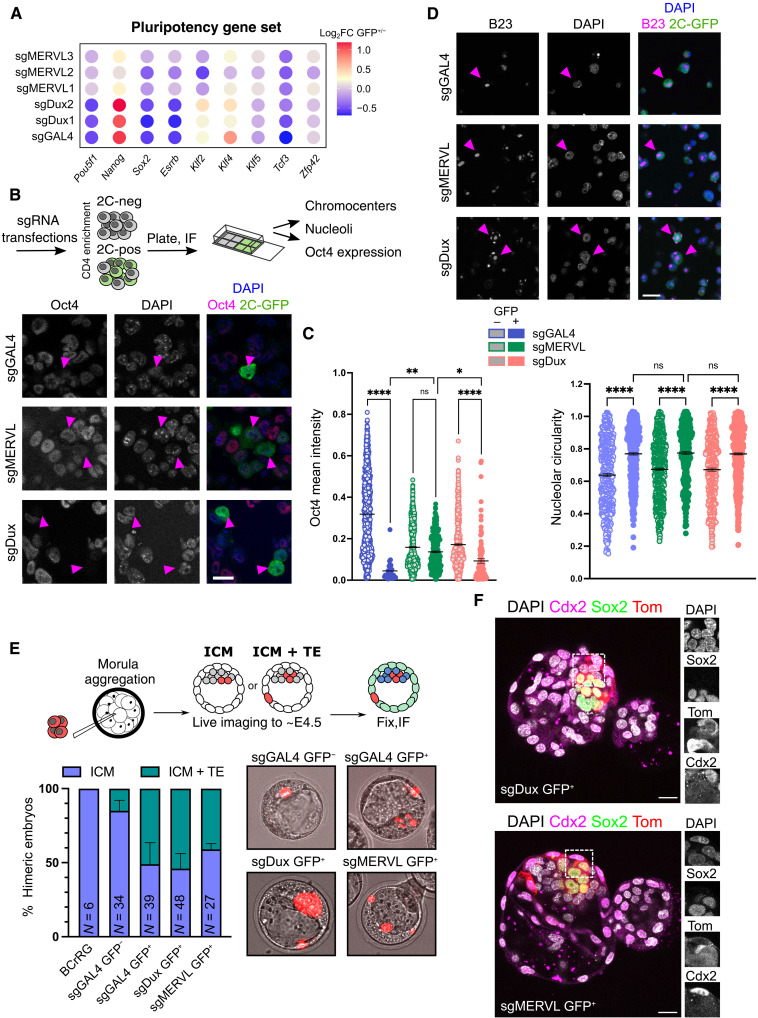
MERVL and Dux-induced 2C-like cells show distinct pluripotency features. (**A**) Bubble plot representing the change in expression of the indicated pluripotency factors in GFP-positive over GFP-negative samples. (**B**) Diagram and representative images of immunofluorescence experiments with sgRNA-induced 2C-like cells. Examples 2C-like cells from CD4-based purifications are indicated with pink arrowheads. Data are representative of at least *n* = 2 experiments. Scale bar, 20 μm. (**C**) Quantification of mean Oct4 intensity (left) or nucleolar circularity (right) in ESCs versus 2C-like cells after sgRNA transfection. GFP-negative and GFP-positive cells were separated by CD4-based purification and processed for imaging [(B) and (D)]. Before plotting, data were filtered to exclude outliers based on GFP intensity. *P* values, one-way ANOVA with Sidak’s multiple comparisons test. Data are representative of two to three experiments. (**D**) Example images of nucleoli (B23) staining in 2C-like cells from the indicated transfections, as in (B). (**E**) Diagram and results of ESC embryo injection experiments. A control ESC line (BCrRG) or TdTomato-labeled CVG ESCs transduced with the indicated sgRNAs were sorted and injected into morula and left to develop to ~E4.5 with live imaging to follow ESC incorporation. Contribution to ICM or TE was scored by position. Data are representative of three independent injection experiments (*n* = embryo number). IF, immunofluorescence. (**F**) Representative confocal images of equivalent E4.5 chimeras following injection of GFP^+^ sgMERVL or sgDux transduced cells at the eight-cell stage. Embryos were immunostained for Sox2, Cdx2, and TdTomato (Tom), counterstained with 4′,6-diamidino-2-phenylindole (DAPI). Maximum projection of five *z*-sections. Scale bars, 20 μm.

To examine the functional importance of MERVL-dependent and MERVL-independent gene expression programs, we next turned to the known features of 2C-like cells. 2C-like cells have been shown to model several characteristics of 2C embryos—including chromatin reorganization, down-regulation of pluripotency factors, and, more recently, immature nucleolar precursor body (NPB)-like nucleoli (*16*, *24*, *26*, *40*). 2C-like ESCs induced from all CRISPRa conditions display down-regulation of various pluripotency genes yet more mildly in sgMERVL cells (Fig. 4A). This is also true for Oct4 (*Pou5f1*) protein, with its down-regulation most marked following Dux induction and only partially down-regulated in sgMERVL 2C-like cells (Fig. 4, B and C, and fig. S6, A to C). Conversely, nucleoli, which are reprogrammed to resemble NPBs in 2C-like cells (*40*), appear similarly NPB-like in all conditions (Fig. 4, C and D, and fig. S6D). Third, we looked at chromocenter decompaction, which is a marker of the more decondensed chromatin state in 2C-like cells and 2C embryos and accompanied by up-regulation of major satellite expression (*24*, *47*, *48*). Notably, the expression of major satellite RNA, GSAT_MM, is not up-regulated by sgMERVL but robustly activated in control and Dux-driven 2C-like cells (Fig. 2C). 2C-like cells driven by sgMERVL show reduced chromocenter decompaction (Fig. 4B and fig. S6E). Together, our results suggest that MERVL expression drives only some of the characteristic features of 2C-like cells.

In culture, 2C-like cells are in a totipotent-like state and, unlike ESCs, can contribute to both embryonic and extraembryonic lineages when injected into morulae (*16*, *27*, *28*). We next investigated whether there are differences in the expanded fate potential of endogenous, sgDux- or sgMERVL-induced 2C-like cells. CVG ESCs were labeled with tdTomato and transduced with sgRNAs (fig. S6F), and then tdTomato^+^/GFP^+^ cells were sorted and injected into wild-type (WT) morulae (Fig. 4E). 2C-GFP–negative cells, i.e., in an ESC state, were found in the inner cell mass (ICM), while control 2C-like cells are present in both the ICM and trophectoderm (Fig. 4E), as previously shown (*27*). Both sgDux and sgMERVL-induced 2C-like cells colonize the ICM and trophectoderm with similar efficiency (Fig. 4E). Contribution to both lineages was confirmed with costaining for Sox2 or Cdx2, respectively (Fig. 4F and fig. S7). Therefore, despite the differences in TEs, gene expression, and 2C-like characteristics in MERVL-induced 2C-like cells, these cells have an expanded fate potential that mirrors cells of the 2C embryo.

### Dux drives cell death independently of MERVL

**Fig. 5.**
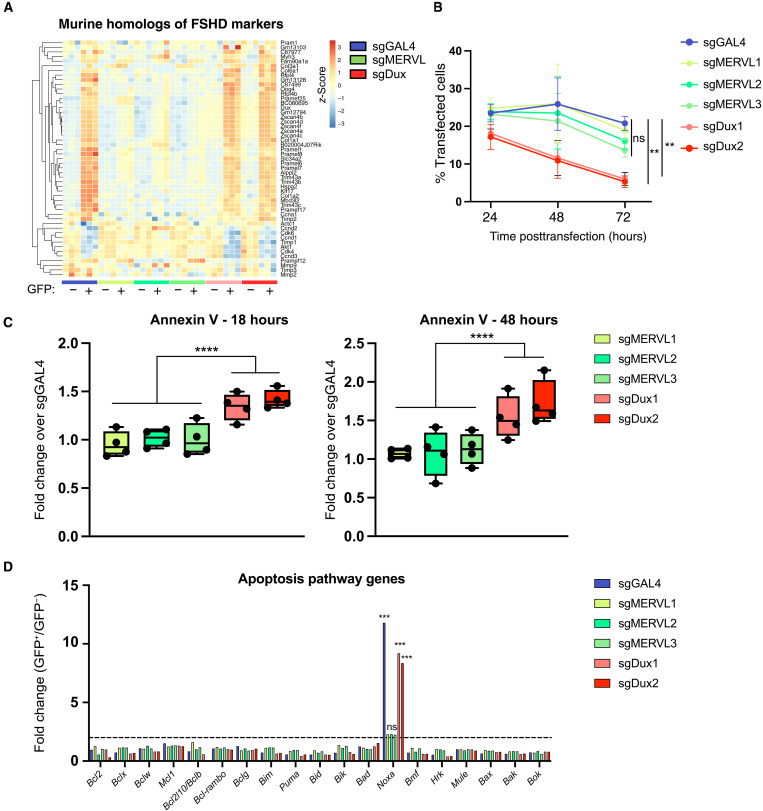
Dux-specific induction of FSHD markers and cell death in ESCs. (**A**) Heatmap of mouse homologs of FSHD marker genes in all GFP-positive and GFP-negative samples. Genes but not samples are grouped by unsupervised hierarchical clustering. (**B**) Histogram showing the percentage transfected (mCherry-positive) cells remaining at the indicated time points posttransfection. Data are mean ± SEM, *n* = 3 independent samples from separate experiments. **P < 0.005 at D2 and D3, two-way ANOVA comparing each sample within every time point to Gal4 as a control. D2, day 2. D3, day 3. (**C**) Annexin V staining experiments at 18 and 48 hours posttransfection with the indicated sgRNAs, quantified by flow cytometry. ****P < 0.0001, two-tailed Mann-Whitney test with *n* = 4 independent experiments. (**D**) Histogram showing log_2_FC in the indicated apoptosis genes in GFP-positive over GFP-negative samples. ***P < 0.001, Toptable FDR.

While the Dux-MERVL axis promotes totipotency and ZGA, it has also been shown that prolonged or excessive activation can affect cell survival in vitro and promote embryo arrest in vivo (*13*, *35*, *39*, *40*). Aberrant DUX4 activation in muscle cells induces an embryonic gene expression program and ERVL family TE activation and drives cell death in FSHD (*32*, *49*). An unanswered question is whether Dux-driven cell toxicity is related to its ability to activate MERVL elements or MERVL-driven genes. Essentially, is MERVL overexpression itself toxic, per se? The downstream mediators of Dux/DUX4-driven cell death are moreover still poorly defined, for example, in FSHD. We asked whether FSHD signatures are induced by sgMERVL and examined known FSHD marker gene homologs in our RNA-seq data. We observed that most are specifically up-regulated in ESCs by sgDux, but not sgMERVL (Fig. 5A). This points to a MERVL-independent network downstream of Dux that is potentially linked to pathology. Tracking CVG ESCs after sgRNA transfection revealed a significant loss of sgDux-transfected CVG ESCs from culture more rapidly than control or sgMERVL-transfected ESCs (Fig. 5B). Dux activation is also correlated with a decrease in several cell cycle regulators, which are also known to be affected in FSHD (Fig. 5A), and we found that ESCs transfected with Dux sgRNAs show mild cell cycle alterations (fig. S8A).

We focused next on a potential link between Dux and apoptosis. Annexin V staining revealed that sgDux-transfected ESCs show increased numbers of apoptotic cells compared to MERVL and GAL4 sgRNAs, as early as 18 hours posttransfection (Fig. 5C). We therefore searched for transcriptional alterations in apoptosis-associated effectors (Fig. 5D). While most pro- or antiapoptotic factors show no clear change, the proapoptotic sensitizer, *Pmaip1*/*Noxa* (*50*), referred to as *Noxa* hereon, is significantly up-regulated in Dux-driven 2C-like cells (Fig. 5D and fig. S8B). Moreover, no significant *Noxa* induction is apparent in 2C-like cells upon MERVL activation (Fig. 5D). Thus, Dux activation leads to reduced cell numbers and changes in the apoptosis pathway that are independent of MERVL activation and associated with poor survival of these cells.

### Noxa contributes to Dux-driven apoptosis in ESCs

**Fig. 6.**
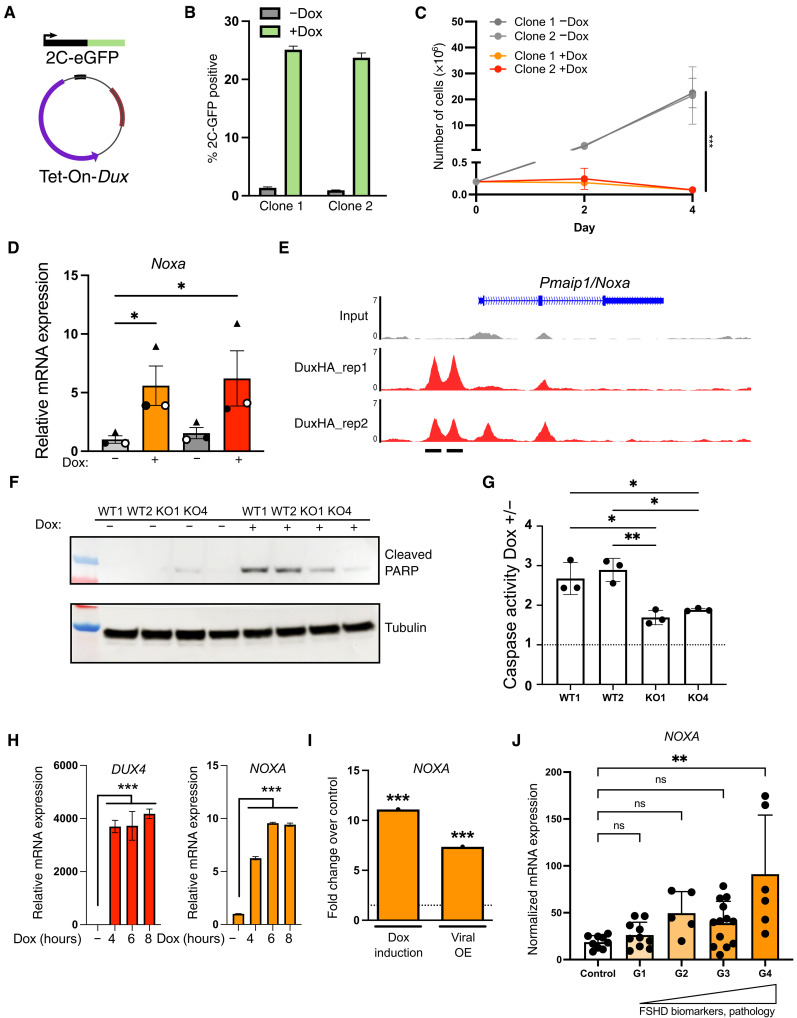
Direct Dux activation of proapoptotic Noxa/NOXA. (**A**) Dox-inducible Dux plasmid, introduced by lentiviral transduction into an independent 2C-GFP reporter ESC line (*13*). (**B**) Histogram showing the percentage of 2C-GFP–positive cells quantified by flow cytometry following Dox-mediated Dux induction. Data are mean ± SD, *n* = 3 independent experiments on two different clones. (**C**) Proliferation of ESCs cultured with or without Dox to induce Dux expression. Data are mean ± SD, *n* = 3 independent experiments, ***P < 0.001, two-tailed Student’s *t* test. (**D**) RT-qPCR of *Noxa* in ESCs following Dux induction. Data are mean ± *n* = 3 independent experiments, *P < 0.05 two-way ANOVA. (**E**) ChIP-seq analysis of the *Noxa/Pmaip1* locus, showing Dux binding at two *Dux* motifs (*51*). ChIP-seq data from (*31*). (**F**) Western blot of WT or *Noxa* KO ESCs 48 hours following Dux induction. Two independent clones, representative of at least *n* = 2 experiments. PARP, poly(ADP-ribose) polymerase. (**G**) Caspase 3/7 assay on *Noxa* WT or KO ESCs 24 hours after Dox induction of Dux. Data are shown as fold increase in activity of Dox-treated over untreated cells, *n* = 3 independent experiments. *P < 0.05 and **P < 0.005 one-way ANOVA. (**H**) *DUX4* and *NOXA* expression in Dox-inducible DUX4 human ESC (hESC) line (*52*). Data are mean ± SEM, *n* = 3 wells, representative of two experiments. *P* values were calculated using one-way ANOVA with Dunnett’s multiple comparisons test. (**I**) Expression analysis of *NOXA* upon DUX4 induction in two FSHD preclinical myoblast models. ***P < 0.001, Toptable analysis, FDR < 0.05. Data from (*76*). OE, overexpression. (**J**) Expression of *NOXA* in biopsies from control or FSHD patient samples (G1 to G4), stratified according to increasing DUX4 target expression and pathology. **P < 0.01, Kruskal Wallis test with Dunn’s correction for multiple comparisons. Data and stratification are from (*53*).

The induction of *Noxa* suggested that it may be a potential mediator of Dux-driven apoptosis in ESCs. To test this, we first established a Tet-ON system to allow for inducible Dux expression (iDux) in an independent 2C-GFP line (Fig. 6A) (*13*). Doxycycline (Dox)–mediated Dux induction leads to robust induction of 2C-like cells by 24 hours, as expected (Fig. 6B and fig. S8C), and these cells are lost from ESC culture in a similar manner to Dux CRISPRa ESCs (Fig. 6C). *Noxa* is up-regulated upon Dux induction (Fig. 6D), and chromatin immunoprecipitation sequencing (ChIP-seq) analysis revealed that Dux binds at two *Dux* motifs upstream of the *Noxa* promoter (Fig. 6E) (*51*). In contrast, no MT2_Mm is found within 50 kb of *Noxa.* These data reveal that the apoptotic factor *Noxa* is a direct Dux target in ESCs.

Noxa is a proapoptotic sensitizer, and it has been shown that altering the balance between levels of pro- and antiapoptotic factors can push cells into apoptosis (Fig. 5D). To test whether Noxa might contribute to Dux-induced apoptosis, we generated *Noxa* KO clones in iDux 2C-GFP ESCs (fig. S9, A and B). We examined markers of apoptosis upon Dux induction in WT and KO clones, revealing that levels of cleaved poly(ADP-ribose) polymerase are lower in the absence of Noxa (Fig. 6F). This is supported by reduced caspase 3/7 activity and increased cell numbers in *Noxa* KO ESCs upon Dux activation (Fig. 6G and fig. S9C). Moreover, Dux-induced apoptosis is partially reduced in Noxa KO ESCs, but not upon rescue with WT Noxa (fig. S9D). These data suggest that Noxa is a Dux-induced mediator of apoptosis and may be partly responsible for the poor survival of Dux-induced 2C-like cells.

Last, we investigated whether the Dux-Noxa relationship is conserved in human cells. We took advantage of recently described iDUX4 human ESCs (hESCs), where the Dux homolog, DUX4, is induced upon Dox addition (*52*). A short pulse of Dox efficiently activates DUX4 target genes that are detectable 12 hours following Dux induction; however, no up-regulation of *NOXA* was seen (fig. S9E). These hESCs also do not appear to undergo extensive cell death (fig. S9F). We tested whether sustained DUX4 expression might instead induce *NOXA* and found that both *DUX4* and *NOXA* expressions are significantly up-regulated by 4 hours following continuous Dox induction, with *NOXA* levels peaking at 12 hours (Fig. 6H and fig. S9G). In agreement, these hESCs exhibit signs of cell death and apoptosis by 12 to 14 hours of Dox treatment (fig. S9F). *NOXA* is also significantly up-regulated in two preclinical myoblast models of FSHD (Fig. 6I). Moreover, in RNA-seq data generated from FSHD muscle biopsies (*53*), *NOXA* expression correlates with DUX4 target expression and is significantly up-regulated in FSHD samples with the highest *ZSCAN4* expression and pathology scores (Fig. 6J and fig. S10, A and B). Together, our results reveal that Dux alone induces cell death in ESCs independently of MERVL and suggests that Dux/DUX4-driven activation of NOXA may be a conserved axis contributing to apoptosis in mouse and human cells.

## DISCUSSION

The transient up-regulation of MERVL transposons is a known, notable feature of murine ZGA*.* It also typifies the 2C-like cells in vitro that have been found to resemble the 2C embryo. Despite this, the mechanisms and functions of MERVL activation are still poorly understood. The relationship between MERVL and its transcriptional activator, Dux, is also a puzzle. Dux expression is both sufficient and necessary for 2C-like cell induction and spontaneous appearance within ESC culture (*31*, *34*). However, multiple groups have demonstrated that *Dux* mzKO embryos are ultimately viable, with sustained MERVL and 2C gene expression (*33*–*35*, *54*). It was thus unclear which parts of this network are the most important for 2C-like features and for development. Conversely, it was also unclear whether the developmental arrest or apoptosis seen upon Dux overexpression in various contexts (*13*, *35*, *40*) is linked to MERVL or due to independent functions of this factor.

Our study sheds light on the Dux-MERVL relationship and its relation to 2C-like characteristics and Dux-induced toxicity. Using a CRISPRa system, we have compared and contrasted the functions of these factors. First, we find that MERVL CRISPRa efficiently activates most of the functional MERVL promoters, and yet this comprises only a portion of the endogenous 2C-like transcriptome and repeatome. In contrast, Dux-driven 2C-like cells are remarkably similar to endogenously occurring 2C-like cells in both their TE and gene expression. Our results reveal that the basis of these differences is MERVL independent; genes promoted by MERVL LTRs are efficiently activated under all experimental conditions. Despite the high number of genes and TEs only up-regulated in control and Dux-induced 2C-like cells, we suggest that these are less important in ZGA. Such MERVL-independent genes are not affected upon *Obox* deletion, which induces early embryo arrest and defective ZGA (*36*). In contrast, MERVL-dependent 2C genes are significantly down-regulated in *Obox* mzKO and are most highly activated in experiments overexpressing Obox3 to improve SCNT embryo progression—consistent with a role for these genes in the early embryo. Together, our data define a minimally required set of 2C genes, driven by MERVL elements. We suggest that a key function of the MT2_Mm promoter is as an integrator of multiple transcription factors (e.g., Obox and Dux), providing several ways of inducing these 2C genes for ZGA.

At a molecular level, MERVL-induced 2C-like cells display similar immature nucleolar morphology as endogenous 2C-like cells. However, they only partially down-regulate Oct4 and fail to decondense chromocenters or up-regulate major satellite RNA, suggesting only a partial reprogramming of 2C-like chromatin. Nevertheless, both MERVL- and Dux-induced 2C-like cells can contribute to ICM and trophectoderm when injected into morulae—a key functional characteristic of the 2C-like state that mimics totipotency in the 2C embryo. Future experiments may shed light into the mechanisms and functions of Dux-driven chromatin reprogramming in these distinct scenarios.

**Fig. 7.**
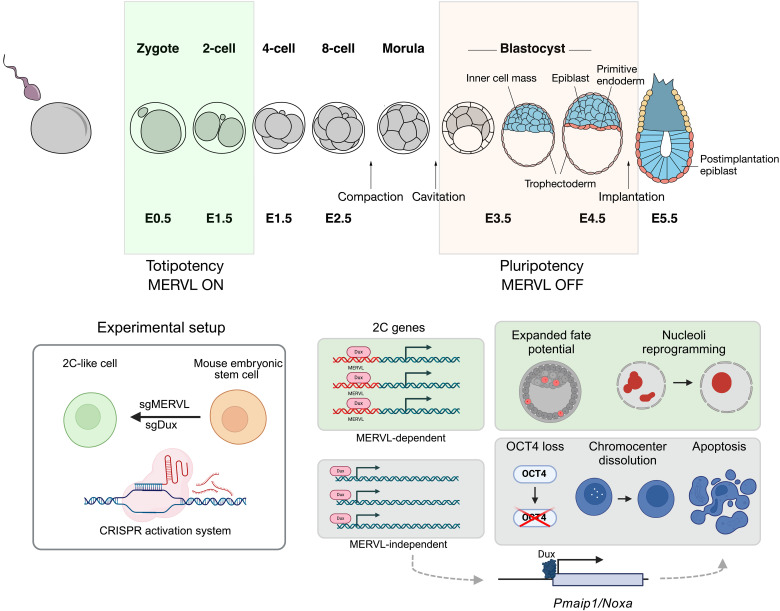
Model showing the different contributions of MERVL-driven or MERVL-independent genes to totipotency features. The totipotent 2C stage of embryonic development is characterized by a short burst of expression of MERVL elements. These elements can act as cis-regulatory regions to induce the expression of nearby genes. We show that MERVL sequences form a landing pad for 2C regulators to induce the expression of a fraction of the 2C transcriptional program. The activation of MERVL-driven 2C genes is enough to lead to nucleolar reprogramming and expanded cell fate potential in ESCs, but they do not drive other key features of 2C-like cells such as loss of Oct4 protein and chromocenter dissolution. In addition, apoptosis induced upon long-term induction of the 2C-like state is driven by Dux and Noxa but independent of MERVL. Illustration created in BioRender. Chammas, P. (2025) https://BioRender.com/b32yjws and https://BioRender.com/jkqpmq5.

Our results build on previous studies that have identified factors that can induce 2C-like and totipotency characteristics (*27*, *28*, *55*), either upstream or downstream of Dux (*56*). Here, through direct comparison, we reveal that direct MERVL or Dux up-regulation confers distinct features of totipotency. Moreover, by defining genes and repeats specific to each part of the axis, our data point to a minimal set of MERVL targets responsible for these totipotency characteristics (Fig. 7). Investigating these factors in future may reveal previously unidentified proteins important in development for essential processes such trophectoderm specification and ZGA.

This work, as well as others, points to some interesting differences between ESC models of the 2C stage and 2C embryos. For example, not all 2C-like genes are expressed in 2C embryos. Moreover, although Dux potently activates both MERVL-dependent and MERVL-independent genes in 2C-like cells, these are not Dux dependent in embryos. The reports of viable *Dux* KO mice have led to suggestions that studying this factor—or 2C-like cells—may have limited interest or utility in development. However, we argue that this dichotomy serves to highlight the multipronged mechanisms in place to ensure ZGA occurs. A system where multiple factors all activate the same core genes provides the opportunity for redundancy and robustness in an essential developmental process. Previously unidentified ZGA factor Nr5a2 activates itself as well as other nuclear receptors such as Esrrb that bind the same motif, up-regulating 75% of ZGA genes (*37*). Multiple Obox factors are expressed in one-cell and 2C embryos, and Obox4 compensates for the loss of Dux (*38*). This raises the question of whether Obox and Dux act truly redundantly or if additional factors present in embryos, but missing from ESCs, further promote MERVL gene expression. Differences in both chromatin organization and factor availability may underlie contrasting dependencies in 2C-like cells versus 2C embryos. For example, both Nr5a2 and Esrrb bind distinct sets of enhancers in 2C embryos and ESCs (*37*). Investigating these discrepancies will shed further light on the mechanisms of ZGA and early development.

The flip side to the Dux-MERVL axis is that its activation is only tolerable for a very short period. Ectopic *Dux* overexpression halts embryo development (*35*), while preventing Dux repression by various means is sufficient to cause 2C arrest (*13*, *40*, *57*). In ESCs, Dux also induces DNA damage at CTCF motifs (*39*). However, the role of MERVL in Dux-dependent cell death was previously unknown. Here, we demonstrate that Dux-dependent cell death is MERVL independent. In addition, we identify the apoptotic sensitizer, Noxa, as a direct Dux target (Fig. 7). *Noxa* deletion reduces Dux-dependent apoptosis in ESCs, and we show that DUX4 also induces *NOXA* in hESCs. Moreover, *NOXA* is up-regulated in preclinical models of FSHD and in FSHD muscle biopsies. These data suggest NOXA may be a promising therapeutic target to reduce myoblast death and, potentially, muscle loss in FSHD. This could function either as a standalone treatment or, more likely, in conjunction with future therapies that could directly repress DUX4 expression (*58*) or those that might inhibit DUX4 signaling but that have not themselves reduced apoptosis (*59*). Of note, *Noxa* KO in mouse ESCs only mildly rescued cell numbers (fig. S9C), in agreement with the likelihood that many aspects of Dux/DUX4-dependent toxicity contribute to poor cell growth and survival.

Given the above, an intriguing question is how Dux/DUX4 expression is tolerated in normal development in both mice and humans. We hypothesize that this is because its expression is only transient in these biological contexts. The 2C-like state is also temporary in cell culture, with endogenously arising 2C-like cells soon transitioning back into ESCs (*16*, *25*). Thus, although Noxa is up-regulated in 2C-like cells (Fig. 5C), this may be brief enough to avoid triggering cell death. Such transience may also be important to avoid other aspects of Dux-dependent pathology, for example, cell cycle defects or DNA damage (*39*). In hESCs, *NOXA* levels only rise after sustained DUX4 activation, unlike other targets such as *ZSCAN4* that are detected after a brief pulse of DUX4. The induction of Noxa/NOXA and so apoptosis may serve as a natural mechanism to prevent prolonged activation of these pathways. Learning more about the processes responsible for totipotency and ZGA, as well as those that trigger pathology, will enhance our understanding of healthy development.

## MATERIALS AND METHODS

### Cell culture

Mouse ESCs were cultured on 0.2% gelatine-coated (G1393-100ML, Sigma-Aldrich) dishes in Dulbecco’s modified Eagle’s medium (DMEM) + GlutaMAX (31966047, Gibco) supplemented with 10% fetal bovine serum (FBS; A5256801, Gibco), 2-mercaptoethanol (31350010, Gibco), nonessential amino acids (11140050, Gibco), and leukemia inhibitory factor (ESG1107, Millipore). Upon establishment, CVG ESCs were cultured in the presence of blasticidin (10 μg/ml; 11583677, Thermo Fisher Scientific) and G418 (250 μg/ml; 11811023, Thermo Fisher Scientific). For cells inducible by Dox, a final concentration of Dox (1 μg/ml) was used (A4052-APE-1g, Stratech Scientific). Length of induction varied between experiments and is indicated in the respective figure legend.

Dox-inducible DUX4-TetOn hESCs (iDUX4 hESCs) were previously generated from the H9 (WA09) hESC line (*52*). Cells were cultured on Geltrex (A1413302, Life Technologies)–coated six-well tissue culture dishes in Essential 8 medium (A1517001, Thermo Fisher Scientific) at 37°C with 5% CO_2_ and passaged every 3 to 5 days after ~5 min of incubation with 0.5 mM EDTA (15575020, Life Technologies).

To induce DUX4 expression, DUX4-TetOn hESCs were treated with Dox (1 μg/ml) in Essential 8 medium (5% CO_2_, 37°C). For pulsed induction, cells were incubated with Dox (1 μg/ml) for 15 min, washed three times with Essential 8 medium, and then incubated in fresh Essential 8 medium for 12 hours. For continuous induction, cells were exposed to Dox (1 μg/ml) in Essential 8 medium (5% CO_2_, 37°C) for varying durations (0, 4, 6, 8, or 12 hours) before being harvested.

### Cell line generation

CRISPRa activity in E14 ESCs was achieved by safe harbor integration of the dCas9-VPR transgene (*43*) driven by a CAG promoter and linked to blasticidin resistance into the *ROSA26* locus. The dCas9-VPR knock-in vector containing ~500-bp 5′- and 3′-homology arms was linearized by Ahd I digestion and recovered following phenol-chloroform extraction and ethanol precipitation. Ten micrograms of linearized targeting vector + 5 μg of pX458-Cas9-ROSA sgRNA were introduced into ESCs by liposome-based transfection with JetPrime (Polyplus-transfection SA). Following transfection, ESCs were plated on gelatine-coated 10-cm plates at low density to ensure clonal expansion. Forty-eight hours after plating, the media was refreshed to ESC media plus blasticidin (2.5 μg/ml) to initiate selection for recombinants. Following 8 to 10 days of blasticidin selection, individual resistant colonies were isolated and transferred to individual wells of a gelatinized 96-well plate for further expansion and PCR screening. Individual clones were further screened for homologous recombination by PCR genotyping of genomic DNA across the ROSA 5′-homology arms. Heterozygous recombinants were subsequently screened for transcriptional activation activity by RT-qPCR following lentiviral delivery of validated sgRNAs against *Hbb-bh1* and *Ttn* (*60*).

To incorporate the 2C-GFP/CD4 reporter into these cells, a single heterozygous *Rosa26*^dCas9-VPR/+^ clone was targeted with our previously described 2C-GFP–t2a–mCD4 reporter construct (*40*) by electroporation, as previously described. Fifty micrograms of each reporter was linearized with DraIII digestion and cleaned up by phenol-chloroform extraction followed by ethanol precipitation before electroporation. Electroporated cells were plated at low density to ensure that clonal density would be preserved. Electroporated cells were subjected to geneticin/G418 selection (250 μg/ml) 24 hours after electroporation. Selection was maintained 7 to 8 days until individual resistant colonies emerged. A single positive clone with expected 2C-GFP behavior was used for all subsequent experiments (CVG ESC line)

To generate Dox-inducible Dux overexpressing mouse ESCs with a 2C-GFP reporter, lentivirus containing Dox-inducible plasmid construct [pCW57.1-mDux-CA, a gift from S. Tapscott (Addgene, plasmid #99284; http://n2t.net/addgene:99284; RRID:Addgene_99284] was produced in 293T cells and used to infect an independent 2C-GFP reporter ESC line (*13*). Infected cells were selected with puromycin (1 μg/ml). Individual clones were picked and expanded, and their response to Dox induction was tested. Two of the most responsive clones were selected and clonally expanded.

To knock out *Pmaip1/Noxa*, guide RNAs (gRNAs) targeting both ends of the *Pmaip1* gene were cloned into the pX458 plasmid carrying either a blue fluorescent protein (BFP) or green fluorescent protein (GFP) reporter and transfected into Dux-inducible 2C reporter cells (table S1). Double-positive cells were then sorted, and individual clones were picked and screened by PCR for the *Pmaip1* gene deletion.

### CRISPRa gRNA design and cloning

The locations of MT2_Mm family members were extracted from the UCSC RepeatMasker track for mm10, and the corresponding nucleotide sequences were extracted from the genome sequence. Candidate SpCas9 sgRNAs, using an NGG PAM sequence, were identified for these elements using FlashFry (*61*), allowing for no mismatches. The MT2_Mm consensus sequence was downloaded from Dfam (DF000004155) (*62*), and, last, guides with the highest coverage in the genome and with exact matches to the consensus sequence were shortlisted.

Dux sgRNAs were designed using the Benchling CRISPR design tool, inputting the promoter sequence and CDS start of *Dux* (*ENSG00000058537*) as input. The two sgRNAs giving the highest Dux induction in transfection experiments were selected for all experiments in this study. For all transfection and transduction experiments, sgRNAs cloned into Aar I sites (Thermo Fisher Scientific) downstream of a U6 promoter in the lentiviral expression vector mp783 were used, which also contains a separate EF1a-driven PuroR-t2a-mCherry reporter cassette.

### Transfection and lentivirus transduction

Transfections were done using Lipofectamine 2000 (11668027, Invitrogen) in Opti-MEM (31985062, Thermo Fisher Scientific) according to the manufacturer’s instructions. Cells were trypsinized and collected 24 or 48 hours posttransfection depending on experimental design. In experiments to increase the levels of MERVL expression upon sgRNA transfection while maintaining constant total (DNA), ESCs were transfected with a total of 6 μg of plasmid in the following conditions: 6 μg of sgGAL4 plasmid, 4 μg of sgMERVL3, and 2 μg of sgGAL4 (M3), a mix of 1.33 μg each of all three MERVL guides and 2 μg of sgGAL4 (sgMERVL), a mix of 2 μg each of all three MERVL guides (sgMERVL 1.5×), and lastly a mix of 2 μg each of the two sgDux guides with 2 μg of sgGal4 (sgDux). Cells were collected 48 hours posttransfection, and mCherry-positive cells were sorted for RT-qPCR or immunofluorescence experiments.

For lentivirus generation, Lenti-X 293Ts (632180, Takara) were maintained in high-glucose DMEM GlutaMAX (31966047, Gibco), 10% FBS (A5256801, Gibco), 1× MEM nonessential amino acids (11140050, Gibco), and 0.1 mM 2-mercaptoethanol (31350010, Millipore). The day before transfection, Lenti-X 293Ts were seeded on 0.1% (w/v) poly-l-lysine–coated (P8920, Sigma-Aldrich) dishes at a density of 105,000 cells cm^2^ and allowed to adhere overnight. sgRNA-containing plasmids were transfected using Lipofectamine 3000 (L3000008, Thermo Fisher Scientific) with packaging plasmids psPAX2 and pMD2.G. Six hours posttransfection, plates were refreshed with lentivirus packaging media (high-glucose DMEM GlutaMAX, 5% HI-FBS, 1× MEM nonessential amino acids, and 0.1 mM 2-mercaptoethanol). Forty-eight hours posttransfection, the media was collected and centrifuged to remove debris. The supernatant was then passed through a 0.45-μm filter and stored at 4°C*.* Media was refreshed for the cells, and 24 hours later, the media was again harvested as above. Lentiviruses collected on 48- and 72-hour time points were concentrated using the 100-kDa Amicon ultracentrifugal units (UFC810024, Millipore), spun at 1500*g*, and maintained at 4°C. Concentrated lentiviruses were aliquoted and snap frozen. Once thawed, unused portions of the aliquots were discarded.

For lentivirus transduction, 10^5^ cells were plated in a six-well plate coated with 0.2% gelatine. The next day, culture media was changed, and polybrene (8 μg/ml; 7711, Tocris) was added to the media before the addition of 15 μl of concentrated virus. Media was changed every day until cells were trypsinized and collected (48 or 72 hours postinfection depending on experimental design).

For Noxa rescue experiments, lentiviral vectors using a CAG promoter driving the expression of Pmaip1/Noxa (or empty control) were designed on the VectorBuilder website and ordered from that same provider. Lentivirus generation and the following transductions into the Dux-inducible Noxa WT/KO cell lines generated previously were done as described above. Pmaip1/Noxa rescue plasmids also included a blasticidin resistance cassette which was used to select for successfully infected cells.

### Flow cytometry

Cells were trypsinized and washed with phosphate-buffered saline (PBS) before being resuspended in FACS buffer (PBS, 3% FBS, and 1 mM EDTA) and passed through a 35-μm strainer cap. Single-cell suspensions were evaluated on an LSRII Flow Cytometer System (FACSymphony A3, BD Biosciences) equipped with FACSDiva software.

### Preparation of 2C^−^ and 2C^+^ cells for RNA-seq

CVG ESCs were transfected with the different gRNA plasmids using Lipofectamine 2000 (11668027, Invitrogen) according to the manufacturer’s instructions. Culture media was changed the next day. Cells were trypsinized 48 hours posttransfection, and 2C^−^ and 2C^+^ cells (GFP^−^ and GFP^+^, respectively) were isolated by FACS. Cells were then pelleted and RNA purified using the RNeasy Mini Kit (74104, QIAGEN). RNA was sent to Novogene for RNA-seq library preparation and sequencing.

### Quantitative reverse transcription polymerase chain reaction

RNA was isolated using the RNeasy Mini Kit (74104, QIAGEN) according to the manufacturer’s instructions. Up to 1 μg of RNA was used for cDNA synthesis using the High-Capacity RNA-to-CDNA kit (4387406, Thermo Fisher Scientific). qPCR was done using KAPA SYBR FAST Rox Low (KK4622, Merck) on a QuantStudio5 (Applied Biosystems). Quantification of gene expression was achieved by normalizing to *Rpl7*. All primers used for qPCR can be found in table S1.

### RNA sequencing

RNA was purified from cells harvested in three independent CRISPRa and FACS experiments. RNA was purified, and then RNA-seq libraries were prepared by Novogene and sequenced paired-end (PE) 150 bp, with an average of at least 30 million reads per sample. Reads were processed using cutadapt v4.7 (*63*) to remove Illumina adapters, trim, and filter on quality with the settings: -a AGATCGGAAGAGCACACGTCTGAACTCCAGTCA, -A AGATCGGAAGAGCGTCGTGTAGGGAAAGAGTGT, -n 1, -m 31, --nextseq-trim 20, --max-n 0. Processed reads were aligned with HISAT2 v2.2.1 (*64*) to GRCm39 with settings adjusted to report up to 100 multiple alignments for each read pair: --rna-strandness RF --no-discordant --no-mixed --no-unal -k 100 --score-min L,0,-0.66 --pen-noncansplice 20 --dta.

Gene and TE locus–level counts were obtained with TElocal [TEtranscripts (*65*)] using settings to allow distribution of multimapping reads using maximum likelihood estimation: --stranded reverse --mode multi. GRCm39.110 (Ensembl) annotations were used for genes. TE annotations were produced using RepeatMasker v4.1.5 configured with HMMER v3.3.2 (*66*) to scan the GRCm39 genome using Dfam3.7 (*62*) HMM TE models in sensitive mode: -s -no_is -norna -spec “mus musculus.”

Within R v4.3.3, gene count data were used to assess intersample relationships using hierarchal clustering and PCA. In addition, the impact of technical experimental factors was assessed using variance partitioning [variancePartition v1.30.2 (*67*)]. Following these assessments, the remove unwanted variation using replicate/negative control samples method [RUVseq (*68*)] was used to remove the replicate batch effect by subsequently integrating the returned correction factors into the statistical model (table S2).

Gene and TE analyses were performed using DESeq2 v1.40.2 (*69*). TE subfamily analysis was performed by summing the counts from individual TE loci. Within DESeq2, independent hypothesis weighting was conducted to optimize the power of *P* value filtering using IHW v1.28.0 (*70*), and log_2_ fold change (log_2_FC) shrinkage was performed using ashr v2.2-63 (*71*). Multiple correction testing adjustments were optimized by passing false discovery rate (FDR) and log_2_FC thresholds at the point of result generation.

### Reanalysis of published RNA-seq datasets

Where normalized data were not available or where necessitated, published datasets were reanalyzed from raw data downloads. Following read trimming, as above, datasets for TE expression assessment were processed identically (HISAT2 followed by TElocal). Alternatively, following read trimming, where the purpose was solely to assess gene expression, transcript expression was quantified directly using Salmon v1.10.1. Both TElocal and Salmon produce transcript abundance estimates using expectation maximization. As for TElocal, the Salmon gene transcript database was constructed using GRCm39.110 (Ensembl) annotations, and downstream processing in R using a DESeq2 workflow was performed identically.

In R, module score assessments were performed using a function repurposing the AddModuleScore() function from Seurat (*72*) to work with bulk RNA-seq data (*73*) (https://github.com/HerpelinckT/geneset-modulescoring).

### MACS immunofluorescence

Magnetic-activated cell sorting (MACS) cell separation was performed using the EasySep Mouse CD4 Positive Selection Kit II (18952A, STEMCELL Technologies). Briefly, CVG ESCs were trypsinized, washed once with PBS, and resuspended in PBS FACS buffer (PBS, 3% FBS, and 1 mM EDTA) at a concentration of 10^8^ cells/ml. 2C-negative (flowthrough) and 2C-positive (beads) cells were purified according to the manufacturer’s protocol and plated on Matrigel-coated chambered slides (354108, Falcon) for immunofluorescence experiments. One hour after plating, cells were fixed for 10 min using 4% paraformaldehyde in PBS. For immunofluorescence, cells were washed with PBS and then blocked and permeabilized using a blocking buffer [PBS, 10% donkey serum, and 2.5% bovine serum albumin (BSA)] supplemented with 0.4% Triton X for 30 min at room temperature. Primary antibodies in blocking buffer were added for 1 hour at room temperature or overnight at 4°C (table S1). After the incubation with the primary antibodies, cells were washed three times with PBS and then incubated with the corresponding secondary antibodies for 1 hour at room temperature. Cells were lastly washed three times with PBS, before the slides were covered with Vectorshield plus 4′,6-diamidino-2-phenylindole (DAPI), mounted, and sealed. For graphs in Fig. 4C, to correct for low enrichment following MACS, outliers were removed on the basis of the unexpected GFP level (e.g., GFP-negative cells were removed from the 2C-positive condition). See also fig. S5A (unfiltered Oct4 data).

### Western blot

Cells were lysed in radioimmunoprecipitation assay buffer [150 mM NaCl, 5 mM EDTA, 50 mM tris (pH 8.0), 1% NP-40, 0.5% sodium deoxycholate, and 0.1% SDS]. Protease inhibitors (78429, Thermo Fisher Scientific) were added freshly before every use. After the addition of 4× Bolt lithium dodecyl sulfate (LDS) sample buffer (B0007, Invitrogen), samples were heated for 5 min at 95°C and loaded on a 4 to 12% tris-glycine gel (XP04122BOX, Invitrogen). The transfer onto a polyvinylidene difluoride membrane (IB24001, Invitrogen) was done using the iBlot 2 (Invitrogen) following the manufacturer’s instructions. Membranes were blocked in 5% nonfat milk (42590.02, Serva) in tris-buffered-saline plus tween (TBST) for 1 hour and incubated with primary antibodies (table S1) overnight at 4°C. Blots were washed three times with TBST and incubated with the corresponding horseradish peroxidase–conjugated secondary antibodies (table S1) for 1 hour at room temperature in TBST. Blots were washed three times in TBST and imaged using the ImageQuant 800 (Amersham) following the manufacturer’s instructions.

### Embryo micromanipulation

Animal studies were authorized by a UK Home Office Project Licence under the Home Office’s Animals Act 1986 and carried out in a Home Office–designated facility. B6CBA-F1 females (6 to 9 weeks old) were superovulated with 5 IU of pregnant mare serum gonadotropin (PMSG) intraperitoneal injection followed by 5 IU of hCG intraperitoneal injection 48 hours after. Preimplantation embryos were collected at embryonic day 2.5 (E2.5) and cultured in potassium simplex optimized medium (KSOM) medium (MR-101-D, Sigma-Aldrich) at 37°C and 5% CO_2_ until micromanipulation. For micromanipulation, E2.5 mouse embryos were placed in prewarmed M2 medium (M7167, Sigma-Aldrich) under light mineral oil (ES-005-C, Sigma-Aldrich). Embryo cell injections were performed on an inverted microscope (Leica DMi8) using Narishige micromanipulators and hydraulic microinjectors. Eppendorf capillaries were used during the micromanipulator for embryo holding (VacuTip I, 5195000036, Eppendorf) and for cell injection [TransferTip (ES), 5195000079, Eppendorf]. Five to six ESCs were injected per E2.5 mouse embryo. After microinjection, embryos were incubated in KSOM medium (MR-101-D, Sigma-Aldrich) at 37°C and 5% CO_2_. A control ESC line constitutively expression tdTomato and OCT4-GFP (labeled as BCrRG) shown to give good ICM contribution (*74*) was used as a control. Images were scored blind by one person, confirmed for selected datasets by a second, and discounted where ambiguous.

### Live-cell imaging of preimplantation chimeras

Time-lapse imaging was performed on an Olympus IX83 microscope equipped with a Hamamatsu ORCA-Flash 4.0 camera, and the environmental chamber was kept at 37°C with a 5% CO_2_ supply. Z-stacks were collected with a step size of 5 μm, every 2 hours for 48 hours using a UCPlanFN N 20×/0.70 objective for embryos. Embryos were placed into microinsert four-well fultrac dish (80406 or 80486, Ibidi) in KSOM medium (MR-101-D, Sigma-Aldrich). Embryos were scored at the blastocyst stage immediately before the moment of hatching for contribution of TdTomato^+^ ESCs to either ICM and/or TE, based on position.

### Immunofluorescence imaging of preimplantation chimeras

Embryos were fixed in 4% paraformaldehyde in PBS for 20 min at room temperature, followed by three 5-min washes in PBS containing 0.1% polyvinyl alcohol (PVA). Samples were permeabilized for 30 min in 0.5% Triton X-100 in PBS at room temperature and then blocked for 1.5 hours at room temperature in 5% BSA-PBS. Samples were incubated with primary antibodies (1:100 in 5% BSA-PBS; table S1) in 10-μl drops in a humidified Terasaki plate (Greiner Bio-One) overnight at 4°C. Embryos were washed three times in PBS containing 0.1% Tween 20 and 0.1% PVA (PBST-PVA) for 5 min. Samples were incubated in secondary antibodies (1:500 in 5% BSA-PBS) (table S1) in 10-μl drops in a humidified Terasaki plate for 1 hour in the dark. Samples were washed three times in PBST-PVA for 5 min and mounted in Vectashield with DAPI (H-1200-10, Vector Laboratories) under #1 coverslips supported by Dow Corning high-vacuum silicone grease (Z273554-1EA, Sigma-Aldrich) on glass slides. Confocal imaging was performed on a Leica Stellaris microscope with a 40× glycerol objective and acquired in 1-μm Z-stacks.

### Caspase 3/7 assay

A total of 10^4^ ESCs per condition were seeded on 0.2% gelatine-coated 96-well plates. Twenty-four hours later, Dox (1 μg/ml) was added for an additional 24 hours to induce Dux expression. The next day, the caspase 3/7 assay was done using the Caspase-Glo 3/7 Assay System (Promega, G8092) according to the manufacturer’s protocol. Luminescence was read and quantified using the FLUOSTAR Omega (BMG Labtech) plate reader.

### Annexin V staining

A total of 2 × 10^5^ ESCs were plated in gelatine-coated six-well plates and, 24 hours later, transfected with CRISPRa guides as described previously. Eighteen or 48 hours later, cells were trypsinized, washed with ice-cold PBS, and resuspended in annexin-binding buffer [10 mM Hepes, 140 mM NaCl, and 2,5 mM CaCl_2_ (pH 7.4)]. Cells were counted and adjusted to a cell density of 10^6^ cells/ml with annexin-binding buffer. One hundred–microliter aliquots were taken, and 5 μl of annexin V conjugate was added according to the manufacturer’s instructions (annexin V, Alexa Fluor 680 conjugate, Thermo Fisher Scientific, A35109). After 15 min of room temperature incubation, aliquots were topped up to 500 μl with annexin-binding buffer, then placed on ice, and analyzed by flow cytometry.

### Cell cycle analysis

A total of 10^6^ ESCs were plated and, 24 hours later, transfected with of our CRISPRa guides as described previously. Forty-eight hours later, cells were trypsinized, washed with PBS, and resuspended in 300 μl of ice-cold PBS. Cells were fixed by dropwise addition of 700 μl of 100% ethanol while vortexing. Cells were left on ice for 30 min before pelleting at 2000 rpm for 10 min and washed twice with PBS. Cells were resuspended in 500 μl of DAPI/Triton X-100 [0.1% Triton X-100 in PBS, DAPI (1 μg/ml)] and analyzed by flow cytometry at 450/50 linear mode.

### Statistical analysis

All details of statistical analyses are outlined within the relevant figure legend, including test, replicates, and any correction for multiple comparisons.
