# Supplementary material for: CRISPRa-mediated disentanglement of the Dux-MERVL axis in the 2C-like state, totipotency, and cell death
Source: Sci Adv. 2025 Dec 19;11(51):eadu9092. doi: 10.1126/sciadv.adu9092 (PMC12716392; doi:10.1126/sciadv.adu9092)
Supplement: Supplementary file 1 — Figs. S1 to S10 [file sciadv.adu9092_sm.pdf]

Science Advances
AAAS

Supplementary Materials for

CRISPRa-mediated disentanglement of the Dux-MERVL axis in the 2C-like state, totipotency, and cell death

Paul Chammas *et al.*

Corresponding author: Michelle Percharde, m.percharde@lms.mrc.ac.uk

Sci. Adv. **11**, eadu9092 (2025)
DOI: 10.1126/sciadv.adu9092

The PDF file includes:

Figs. S1 to S10

Other Supplementary Material for this manuscript includes the following:

Tables S1 and S2

A

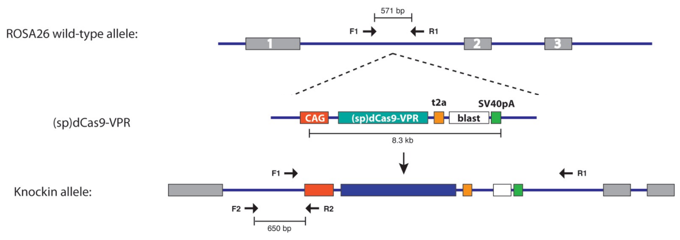

B

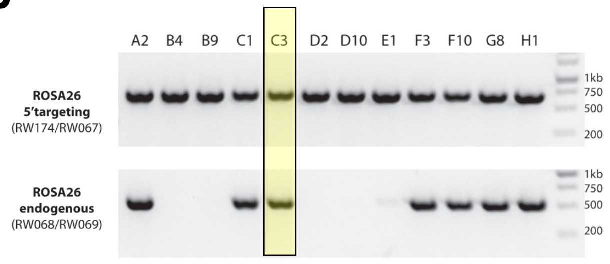

C

MT2_Mm consensus

```
>DF0004155:MT2_Mm
TGTAGTGGCTATTCCTGGTTGTCAACTTGACAATATTTGGAATGAACTACAATCCGGAATTGGAAGGCTCACCAGTG
ACCCTTATCTGGAGGCTTGGAGATCCTTATCTGGATCTTGGTTTGAAGATCTTGAGCCATAGTGGCTATGGATTCCA
GAAGATTGAATCTCCGAGTTTAAGGAACACACCTTTAATCTGGGCTACGCCTTTCATCTGGGATTAAAGGTGTGGTG
GAACACACCTTTAATCTGGGCTACACCTTCTGCTGGAGACAATATAAGGACATTGGAAGAAGGGAGTCTAGCTCTTG
CTCTTGCTCCTTCGCCTGCTTGCTGCGTGAGACTGAGTAACTGCTAGATCCTTGGACTTCCATTCACAGCTGCGACT
GAACAATTGTTGGGAATTGGGCTGCCGACTGTAAGTCATCAATAAATTCCTTTACTATCTAGAGACTATCCATAAGT
TCTGTGACTCTAGAGAACCCTGACTAATACA
```

MERVL sgRNA 1
MERVL sgRNA 2
MERVL sgRNA 3
Mt2_Mm TSS

D

mDux

```
CCCCGTGGCTTGCCAGGGATGAGGAGCCTTTGGGGAGATTTTGCTGAGTGTCAGAGGACGCTTGAGGTCCGCCTCCTGG
TGCCCTGATGTCAGGTGCCAGGCGTGGCAGGCTTGGCGCTTCTTGGGGGCAATGCGAGAAAGGGGTAGGCATGTTCTGT
AGCCCAAGGCGCCTAACTGGTAGGTAGTGGGCGGGACTACCTGAGCAGAGGCAGAGGTATTTAAGGGGCAGTGGTCACA
GCCACTCTGCTGGCAGTTGTTGCAGCTTGTGTTTGTTCTGAAGCTGTCCTGAGTCGAGTCTCCCAAGGTGAGGACTCCT
GGGAGGCCGTCATTGGCACCATG
```

Dux sgRNA 1
Dux sgRNA 2
Dux coding start

Figure S1 dCAS9-VPR cell line and CRISPRa guide selection

A) Diagram of *Rosa26* locus targeting strategy with *CAG-dCas9-t2a-Bsr* construct. Targeting was enhanced by co-transfection with a plasmid containing wt Cas9 plus sgRosa26 guide.
B) Genotyping results for dCas9-VPR ESCs. Clone 3, used for 2C-GFP/CD4 targeting, is highlighted.
C) Consensus sequence of the MERVL LTR, MT2_Mm, showing the positions of *sgMERVL1/2/3.*
D) Sequence of the mouse *Dux* promoter region, with the position of *sgDux1* and *sgDux2* indicated.

Figure S2

Chammas et al.

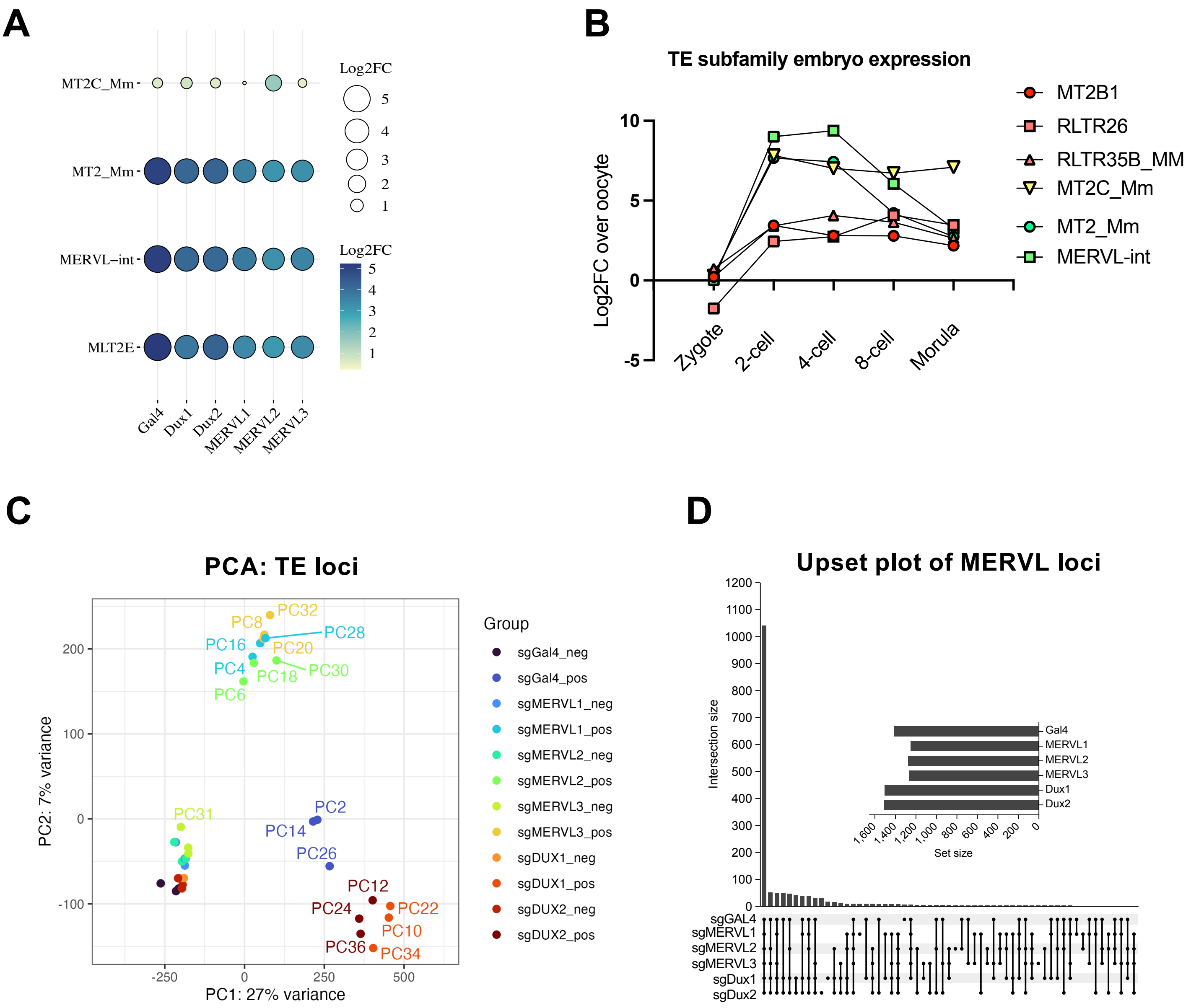

Figure S2 RNA-seq analysis of TE subfamilies and TE loci

A) Bubble plot showing the log2-fold change(FC) in expression in the indicated TE subfamilies in GFP-positive over GFP-negative samples.

B) Expression data showing log2FC of the indicated TE subfamilies in each embryo stage, relative to levels in oocytes. Data from Modzelewski et al., 2021 *(9)*, Xue et al., 2013 *(https://doi.org/10.1038/nature12364)*

C) PCA plot of GFP-positive and negative samples clustered according to expression of individual TE loci. The first two principal components are shown.

D) Upset plot showing overlap in the number of MERVL (MT2_Mm, MERVL-int) loci significantly upregulated by each sgRNA in GFP-positive over negative samples (FDR < 0.05).

Figure S3

Chammas et al.

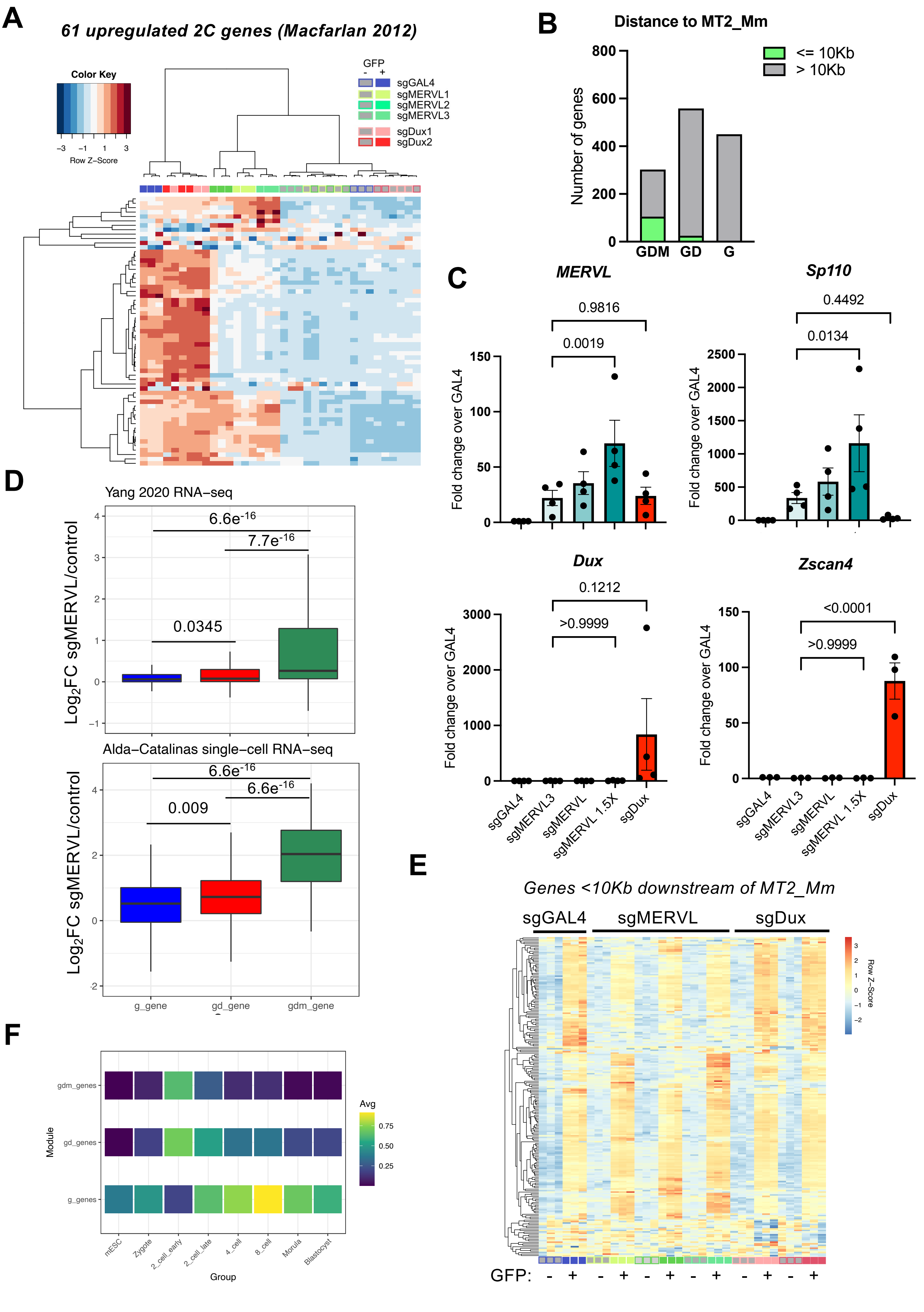

Figure S3 RNA-seq analysis of gene expression data

A) Heatmap showing the expression of 2C-specific genes (Macfarlan et al., 2012 (16)) across all GFP-positive and negative samples. Genes and samples are grouped by unsupervised hierarchical clustering.

B) Histogram showing the number of significantly upregulated genes in GDM, GD or G datasets that are within, or greater than, 10Kb from a MT2_Mm element.

C) Analysis of the indicated genes by RT-qPCR, comparing induction by a MERVL single guide (sgMERVL3), 3 guides combined (sgMERVL), and combined guides at 1.5X amount (sgMERVL 1.5X), alongside sgDux (1X amount). Data are from 3+ independent experiments, relative to sgGAL4. P values, one-way paired ANOVA with Sidak correction.

D) Boxplots showing log2FC of genes within indicated gene sets (G, GD, GDM) in sgMERVL over sgControl CRISPRa experiments from Yang et al., 2020 (28) (GSE119819) and Alda-Catalinas et al., 2020 (44) (GSE135554). P values, Wilcoxon rank-sum test between each condition and sgGAL4, with Bonferroni Correction for multiple comparisons.

E) Heatmap showing the expression of all genes within 10Kb downstream of an MT2_Mm element in GFP-positive and negative samples. Genes, but not samples, are grouped by unsupervised hierarchical clustering.

F) Heatmap of G/GD/GDM gene sets, indicating module expression score for each set at the indicated developmental stages. Data are generated from nascent RNA-seq, Sakamoto et al., 2024 (45) (GSE235547).

Figure S4 **Chammas et al.**

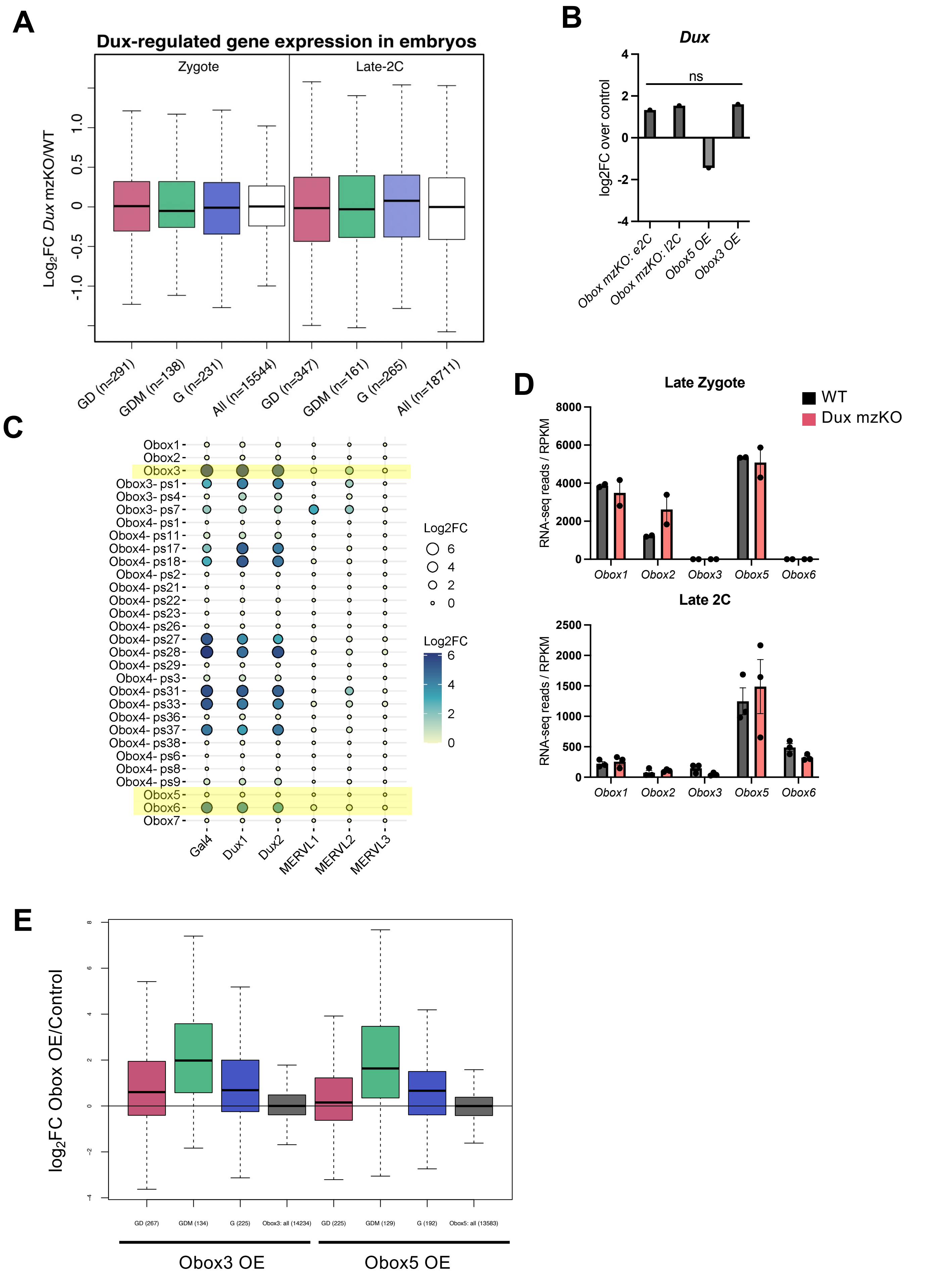

Figure S4 Relationship between Dux, OBOX, and distinct 2C-like gene sets

A) Boxplots showing log2FC of the indicated gene sets (GD, GDM, G, all) in Dux mzKO over WT embryos. Gene sizes are shown in brackets. Data from Chen et al., 2019 (33)

B) *Dux* expression in Obox depleted or overexpressing samples (embryos or ESCs, respectively). Data from Ji et al., 2023 (36), ns = not significant (FDR >0.05)

C) Bubble plot showing log2FC in the indicated *Obox* transcripts in GFP-positive over negative samples from each sgRNA transfection.

D) Histogram showing the normalized expression (RPKM) of *Obox* factors in WT versus *Dux* mzKO embryos. Data from Chen et al., 2019 (33).

E) Boxplots showing log2FC of the indicated gene sets (GD, GDM, G, all) in ESCs upon either Obox3 or Obox5 overexpression. Gene sizes are shown in brackets. Data from Ji et al., 2023 (36).

Figure S5 **Chammas et al.**

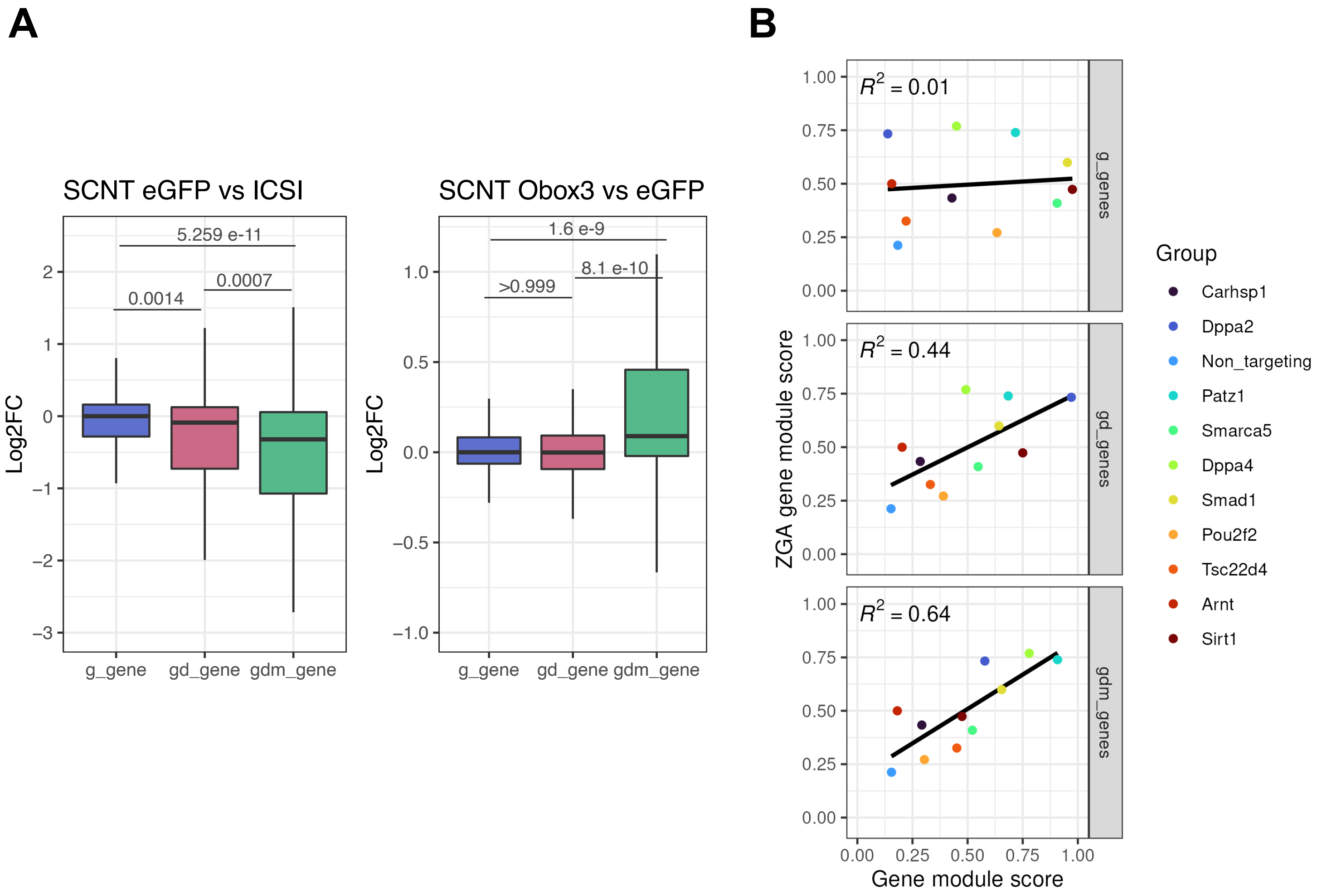

Figure S5 Developmental relevance of MERVL-driven gene sets

A) Boxplots showing log2FC of the indicated gene sets (GD, GDM, G) in embryos generated via SCNT or ICSI, with or without Obox3 overexpression. P values, Wilcoxon rank-sum test between each condition and sgGAL4, with Bonferroni Correction for multiple comparisons. Data are from Sakamoto et al., 2024 (45) (GSE235547).

B) Scatter plots showing the relationship between the gene module score of indicated gene sets (GD, GDM, G) and ZGA module score of factors tested in CRISPRa experiments to promote ZGA-like expression patterns in ESCs. Data are from Alda-Catalinas et al., 2020 (44) (GSE135512). R^2 values of the strength of correlation are shown.

Figure S6

Chammas et al.

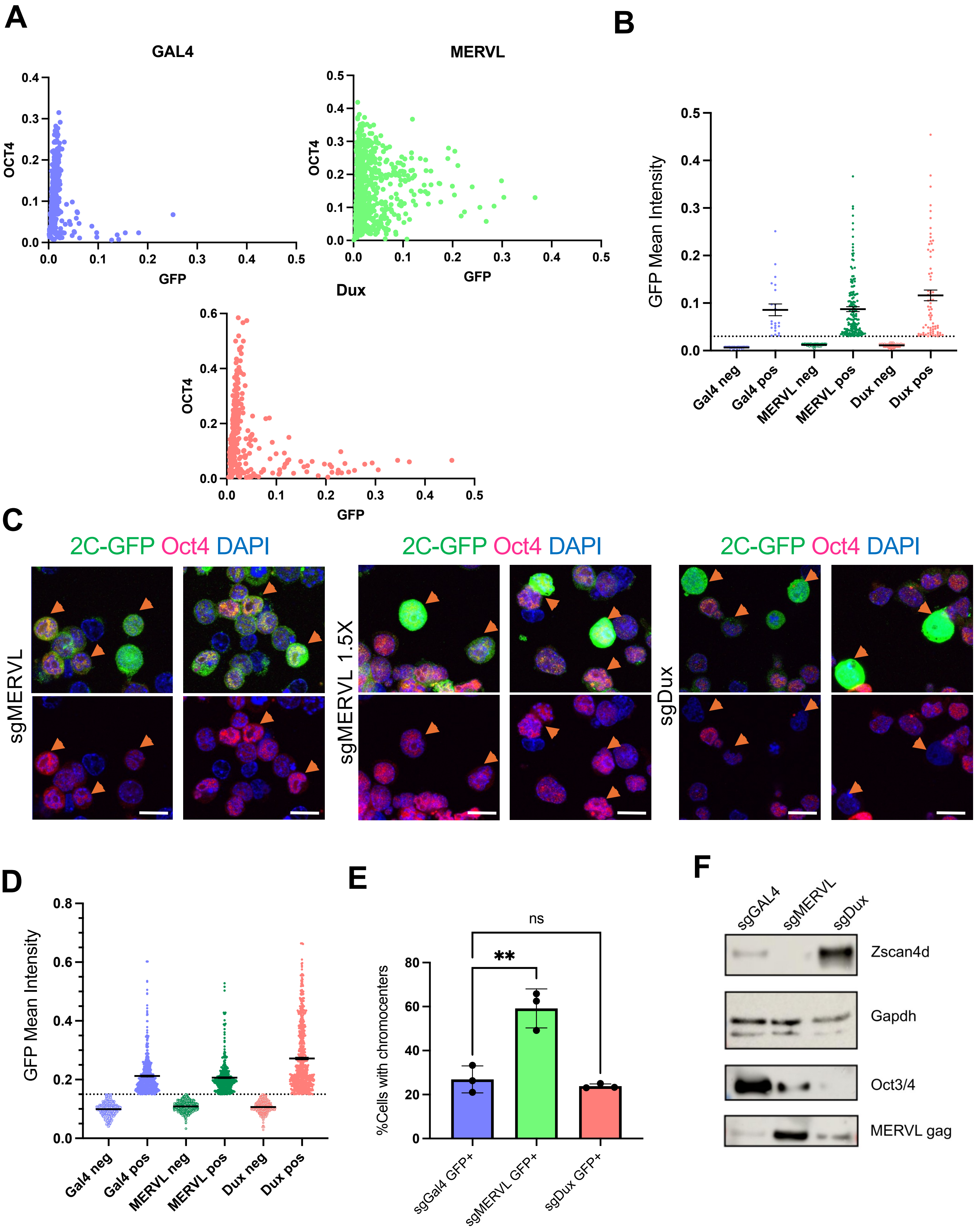

Figure S6 Totipotency features of Dux and MERVL-induced 2C-like cells

A) Scatter plot showing the relationship between the mean intensity of Oct4 and GFP in MACS-sorted cells after indicated sgRNA transfections. Oct4 levels are generally higher in GFP-high cells upon sgMERVL. Data are combined from 2 independent experiments.
B) Graph to indicate GFP mean intensity in cells from MACS purifications used for Oct4 quantifications, showing thresholding level (dotted line) to remove outliers based on GFP.
C) Oct4, GFP and DAPI staining of 2C-like cells (GFP+) in experiments boosting MERVL activation by combining 3 MERVL guides (sgMERVL), and transfecting 1.5X sgRNAs (sgMERVL 1.5X), compared to sgDux guides. Orange arrows highlight example Data are representative of n=2 experiments. Scale, 20μm.
D) Representative graph as in B), but for experiments quantifying nucleolar circularity.
E) Quantification of the percentage of GFP-positive cells from the indicated sgRNA transfections that are chromocenter positive. Data are from 3 independent experiments. P < 0.01, one-way ANOVA with Dunnett's multiple comparisons test.
F) Western blot of samples from unsorted CVG ESCs 72h after transduction with the indicated sgRNAs. Representative of n=2-3 experiments.

Figure S7

Chammas et al.

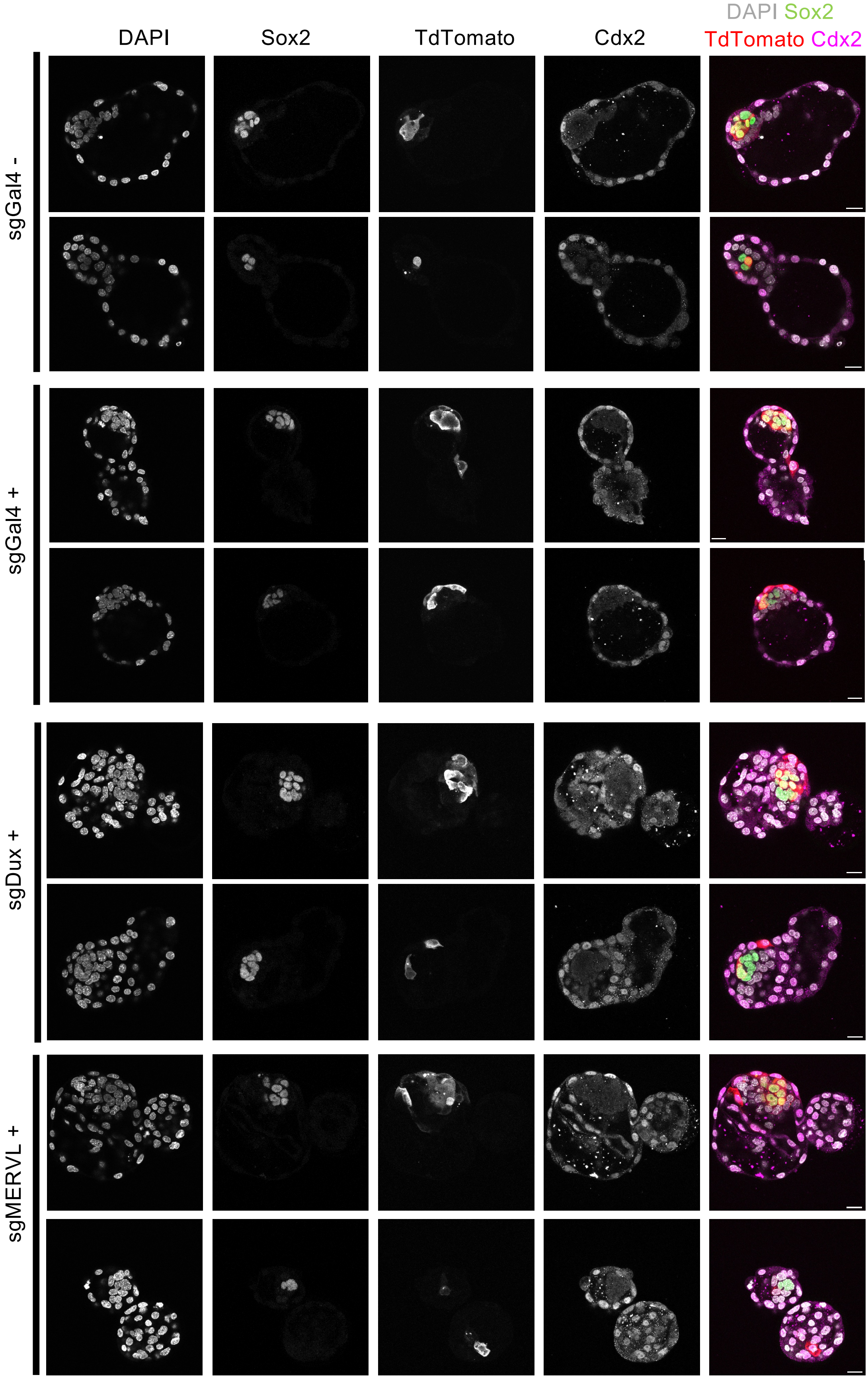

Figure S7 Immunofluorescence of embryo chimeras
Representative confocal images of embryonic day (E) 4.5 blastocysts following injection of sgGal4 GFP-, sgGal4 GFP+, MERVL GFP+ or Dux GFP+ cells at the 8-cell stage. Embryos were immunostained for Sox2, Cdx2, and TdTomato and counterstained with DAPI. Two examples are shown for each condition. For each of sgDux and sgMERVL conditions, one example is the same embryo as shown in Figure 4F. Maximum projection of 5 z-sections of 1 micron. Scale bar = 20um.

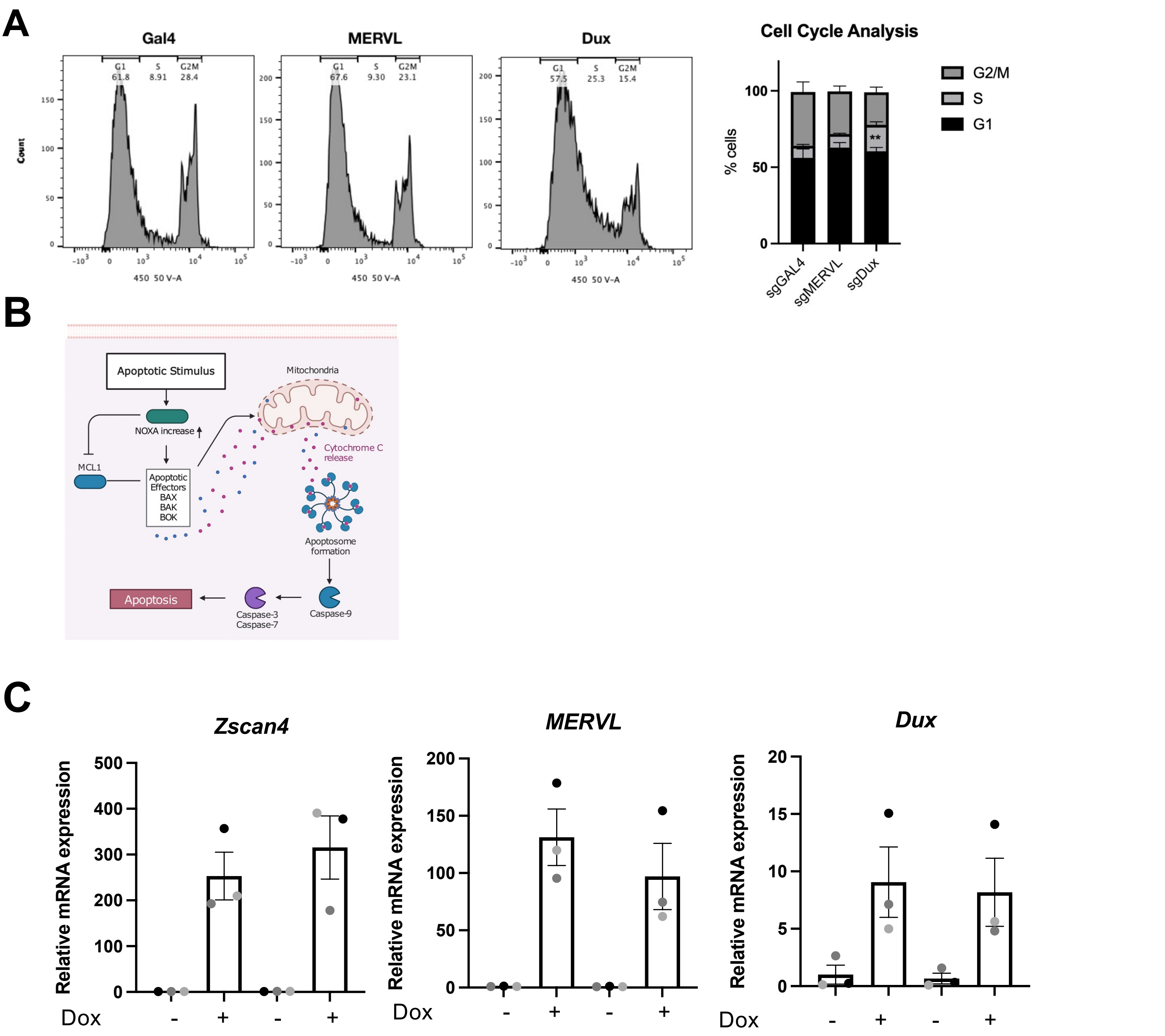

Figure S8 Cell cycle and apoptosis measurements in sgRNA-induced 2C-like cells

A) Cell cycle analysis via DAPI staining for ESCs (mCherry-positive) following transfection with the indicated sgRNAs. Shown are representative flow cytometry histograms and percentages of cells in each cell cycle state. ** P < 0.001, One-way ANOVA with Dunnett's test, comparing S phase percentage in all samples relative to sgGAL4. Data are from n=3 experiments.

B) Diagram showing the role of Noxa as a pro-apoptotic sensitizer in cells. Created in BioRender. Chammas, P. (2025) https://BioRender.com/k44m033.

C) RT-qPCR analysis of the indicated 2C genes in iDux ESCs 24h after dox treatment. Data are mean +/- s.e.m, n=3 independent experiments.

Figure S9 **Chammas et al.**

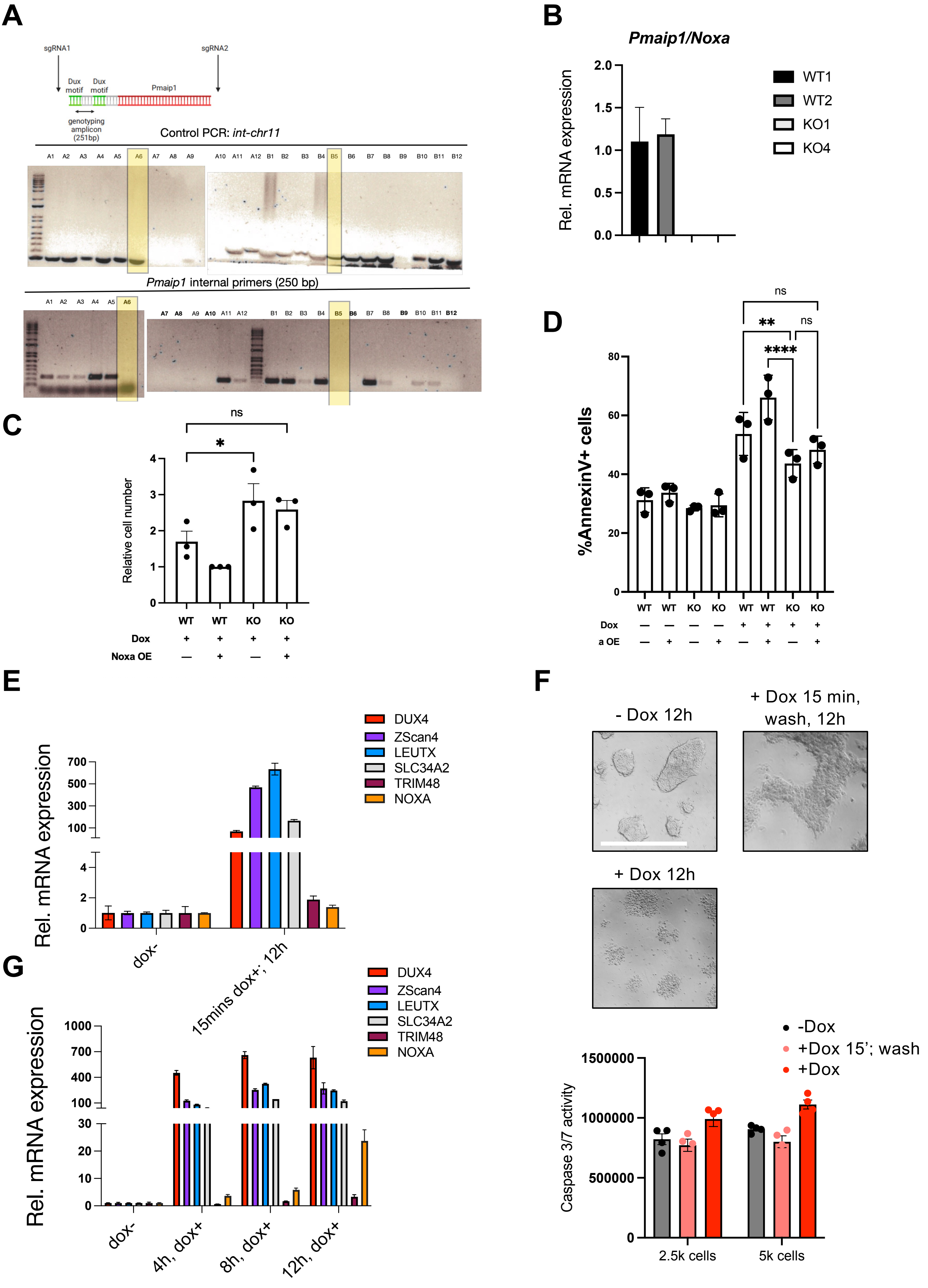

Figure S9 NOXA analysis in mouse and human cells

A) Genotyping PCR strategy and results for the generation of *Noxa* KO mouse ESCs
B) RT-qPCR validation showing no expression of *Noxa* in KO ESCs. Data are mean +/- s.e.m of n=3 independent experiments.
C) Cell count on day 3 after dox-mediated Dux induction in *Noxa* WT or KO mouse ESCs. P values, one-way paired ANOVA with Sidak's multiple comparisons test, from n=3 independent experiments.
D) Quantification of apoptosis via Annexin V staining in dox-inducible Dux ESCs, WT or KO for *Noxa.* ** P < 0.01, **** P < 0.0001, one-way paired ANOVA with Tukey's multiple comparisons test, from n=3 independent experiments.
E) qRT-PCR in human H9 iDUX4 hESCs for the indicated genes. Samples were harvested 12 hours after a 15 minute pulse induction with Doxycycline (dox) to induce DUX4 expression. Data are mean +/- s.e.m of n=3 wells.
F) Representative images and caspase-3/7 assay of iDUX4 hESCs in the absence or presence of Dox for a 15 min pulse or kept on cells constantly. Data are representative of 3 experiments, harvested at 12-14h after Dox. Scale bar, 100 um
G) qRT-PCR expression in H9 iDUX4 hESCs for the indicated genes following increasing duration of continuous Doxycycline-mediated DUX4 induction. Data are mean +/- s.e.m of n=3 wells.

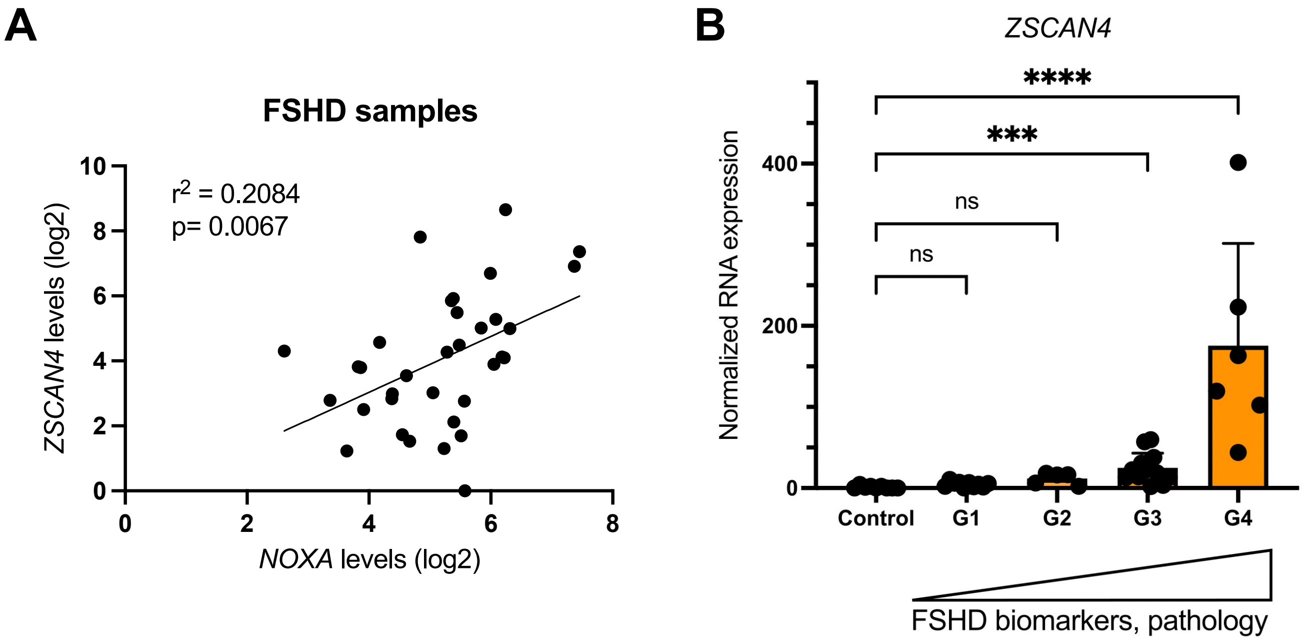

Figure S10 NOXA analysis in FSHD

A) Scatter plot of the log2 normalized expression of *NOXA* versus *ZSCAN4* in FSHD patient samples. Goodness of fit and P value, simple linear regression.

B) Expression of the DUX4 target, *ZSCAN4* in biopsies from control or FSHD patient samples (G1-G4), stratified according to increasing DUX4 target expression and pathology. *** P<0.001, **** P < 0.0001. P values, Kruskal Wallis test with Dunn's correction for multiple comparisons. Data and stratification in A-B are from Wang et al., 2019 (53) (GSE115650).
